# Household coverage of vitamin A fortification of edible oil in Bangladesh

**DOI:** 10.1371/journal.pone.0212257

**Published:** 2019-04-03

**Authors:** Ramkripa Raghavan, Grant J. Aaron, Baitun Nahar, Jacky Knowles, Lynnette M. Neufeld, Sabuktagin Rahman, Prasenjit Mondal, Tahmeed Ahmed

**Affiliations:** 1 Global Alliance for Improved Nutrition (GAIN), Geneva, Switzerland; 2 Center on the Early Life Origins of Disease, Department of Population, Family and Reproductive Health, Johns Hopkins University Bloomberg School of Public Health, Baltimore, United States of America; 3 International Centre for Diarrhoeal Disease Research (icddr,b), Dhaka, Bangladesh; 4 James P. Grant School of Public Health, BRAC University, Dhaka, Bangladesh; TNO, NETHERLANDS

## Abstract

Mandatory fortification of edible oil (soybean and palm) with vitamin A was decreed in Bangladesh in 2013. Yet, there is a dearth of data on the availability and consumption of vitamin A fortifiable oil at household level across population sub-groups. To fill this gap, our study used a nationally representative survey in Bangladesh to assess the purchase of fortifiable edible oil among households and project potential vitamin A intake across population sub-groups. Data is presented by strata, age range and poverty–the factors that potentially influence oil coverage. Across 1,512 households, purchase of commercially produced fortifiable edible oil was high (87.5%). Urban households were more likely to purchase fortifiable oil (94.0%) than households in rural low performing (79.7%) and rural other strata (88.1%) (p value: 0.01). Households in poverty were less likely to purchase fortifiable oil (82.1%) than households not in poverty (91.4%) (p <0.001). Projected estimates suggested that vitamin A fortified edible oil would at least partially meet daily vitamin A estimated average requirement (EAR) for the majority of the population. However, certain population sub-groups may still have vitamin A intake below the EAR and alternative strategies may be applied to address the vitamin A needs of these vulnerable sub-groups. This study concludes that a high percentage of Bangladeshi population across different sub-groups have access to fortifiable edible oil and further provides evidence to support mandatory edible oil fortification with vitamin A in Bangladesh.

## Introduction

Bangladesh has experienced rapid health and nutritional gains in the past few decades. Prevalence of night blindness among pre-school aged children has plummeted from 3.6% in 1982–83 to 0.6% in recent years and these gains are attributable to the implementation of vitamin A supplementation program by the government of Bangladesh [1]. Since 1973, the Bangladesh government has been implementing a national vitamin A supplementation program. In 2013, the bi-annual national vitamin A campaign had a reported coverage of 98% [2]. Despite the success of supplementation programs in reaching the target population, recent national micronutrient status surveys suggest a 20.5% prevalence of vitamin A deficiency in pre-school children [1]. Vitamin A deficiency also remains a significant public health challenge for other population groups such as school-aged children, non-pregnant and non-lactating women [3–5].

Low dietary intake of vitamin A rich foods is one of the main reasons for vitamin A deficiency in Bangladesh, with only 3% of rural women and 7% of rural children assessed have adequate vitamin A intake [6]. Dietary diversity, although desirable, is very hard to attain in a resource-limited setting [7]. A variety of factors including lack of access to diversified diet and fluctuating food prices in Bangladesh, makes it challenging to rely on dietary diversification as a primary strategy [8]. Large-scale food fortification, defined as the addition of micronutrients to commonly consumed staple foods, is a widely used approach to address micronutrient deficiencies [9–11]. Fortification has been repeatedly demonstrated as an effective strategy especially because of its ability to reach populations through existing food-delivery systems without any need to modify dietary habits [12, 13]. Edible oil in Bangladesh is a suitable vehicle for vitamin A fortification because of the advantages such as centralized processing, widespread distribution and high consumption [11]. Further, the benefits of oil fortification have been shown to far outweigh the cost, with benefit to cost ratio estimated to be 50:1; in other words, US$17 to US$22 for every disability adjusted life year saved [14].

In 2013, the government of Bangladesh passed a bill that mandated fortification of edible oil with vitamin A [15] with the intent to complement the existing, targeted, supplementation programs and expand the reach of vitamin A interventions to other population groups besides children 6–59 months of age. Within the first six months after introduction of oil fortification, refineries started fortifying palm and soybean oil with vitamin A. The National Micronutrient Status Survey in 2011, conducted prior to mandatory fortification, provided information on typical edible oil consumption and the associated potential vitamin A intake for specific population groups such as pre-school age children, school age children and non-pregnant and non-lactating women, but there is limited information on other population groups [16]. Having information on the entire population would be important to help provide a baseline from which the overall impact of the program can be monitored. Hence, the purpose of this paper is to utilize a nationally representative survey in Bangladesh to assess the coverage of oil among households and explore the association with potential risk factors, specifically strata, age range and poverty. In addition, this study estimated vitamin A that will potentially be available from fortified edible oil. The survey primarily focused on household access to iodized salt and related knowledge, awareness and purchasing practices with assessment of the type and purchasing practices for household edible oil as its secondary objective [17].

## Subjects and methods

### Study design and setting

The study was a cross-sectional, representative survey of purchase and household use of edible oil in Bangladesh collected as part of the national iodized salt household coverage survey conducted across all 64 districts between January and April 2015. The sampling frame was based on 15,000 primary sampling units (PSU) from the 2009 Multiple Indicator Cluster Survey (MICS)[18], which were re-stratified into three domains for the current survey: 1) urban, 2) rural “low performing,” and 3) rural other [18]. The urban, slum and municipality PSUs, as identified by the 2009 MICS, were combined to constitute the urban stratum. The rural low performing stratum encompassed generally hard-to-reach districts with lower socio-economic status and was identified based on poor indicators for access to iodized salt. All other rural PSU that were not included in rural low performing stratum, constituted the rural other stratum [17]. The selection of primary sampling units within each stratum was based on probability proportional to size methodology [19]. This was followed by a three-stage stratified sample was used to select households. During the first stage, 42 PSU within each stratum were randomly selected. This was followed by the second stage, where a segment of 100 households per PSU were randomly selected. Finally, in the third stage of sampling, a fixed number of 12 households within each PSU were selected using systematic sampling. The final national sample included 1,512 households from 126 PSU nationwide.

### Quality control

The overall quality control of the survey was implemented in four different phases: 1) training and refresher of the field staff; 2) quality control at the field level through supervision and monitoring; 3) ratification of survey methods by the Technical Advisory Committee comprised of experts from the government and other national and international organizations; and finally, 4) quality control of survey data through developing a database with validation checks, cleaning and processing of data.

### Ethics and enrolment of participants

Approval for the study was obtained from the Institutional Review Board of International Center for Diarrhoeal Disease Research, Bangladesh (icddr, b). All adult participants in this study provided written, informed consent. Replacement households were selected when a respondent was not at home or refused to provide consent for the interview. A household questionnaire was used to collect information primarily related to salt iodization, however, with the inclusion of the following information used in relation to data presented in this paper: household characteristics, demographics, information required to calculate a household multidimensional poverty index (MPI) score, the type, source and brand of the main edible oil used by the household, and frequency and quantity of edible oil purchased. For the purposes of this analysis, the following definitions were used. The main edible oil was defined as the oil that the household used on most days in most meals in the home. Fortifiable edible oil included soybean and palm oil, since they are mandated to be fortified by the Government of Bangladesh. Throughout the analysis, fortifiable edible oil is addressed as such and results are not presented by individual oil type. Non-fortifiable oil is defined as edible oil that is not mandated to be fortified (e.g. mustard seed oil) or is homemade.

The MPI, a weighted index that classifies whether a household is in poverty by using component indicators of the three dimensions of education, health, and standard of living, was used in this study to categorize the survey households [20, 21]. The modules to collect information related to iodine deficiency and salt iodization are presented elsewhere [17]. The household member responsible for cooking was preferentially selected as the respondent. In their absence, a woman of reproductive age (15–49 years of age) was selected and if neither of these respondent categories were available, another adult household member present at the time of visit was chosen as the respondent.

Data relating to the type, source, brand, and frequency and quantity of edible oil purchased for each household was collected using a standardized questionnaire (S1 Table). For the purposes of this analysis, it was assumed that household members exclusively consumed edible oil used by the household and the method did not account for oil consumed from processed foods or from food prepared outside the home. Additionally, it was assumed that the oil purchased at the household level was distributed among its members in direct proportion to each member’s share of the household’s total adult male consumption equivalents [3]. Several studies in the literature have used this Household Consumption and Expenditure Survey (HCES) approach to calculate individual consumption based on purchase of foods at household level [22–24]. The process to calculate consumption indicators has been explained in detail in our earlier paper [25]. Briefly, the following steps were used to ascertain potential individual vitamin A intake from the intake of fortifiable edible oil:

Step 1: Overall daily household consumption was estimated based on reported estimates of quantity of oil purchased and time period it takes to consume the amount of oil (daily household oil consumption = quantity of oil purchased (ml)/duration) for all households. Then converted to grams consumption, using a conversion factor of 0.92 g/ml, based on its density [26].

Step 2: Individual daily oil consumption was assessed based on the adult male equivalent (AME) method, which is an aggregate indicator for the household that uses adult males as benchmark for comparison. The AME for different population groups was calculated using an algorithm [23]. Briefly, an age- and sex-specific adult male equivalent (AME_Person_) was assigned to each member of the household over 12 months of age. Then, the AME_Person_ value of each household member was summed up to calculate the household adult male equivalent (AME_HH_). Finally, individual oil consumption was estimated as a ratio of AME_Person_ and AME_HH_ multiplied by the overall daily household oil consumption in grams for that household. Fortifiable oil consumption for children < 12 month of age was not calculated under the assumption that vitamin A from fortifiable edible oil may be very low for this age group.

Step 3: Accounting for vitamin A lost in storage and cooking: Stability of vitamin A depends on a variety of factors including oxidative stability, packaging type, storage conditions and initial oil quality [27]. A recent study by Pignitter et al. estimated that soybean oil stored under household conditions resulted in a decrease of serum retinol concentration of 1.46% to 2.87% per day [28]. Households in our study sample purchased oil every 14.2 days, potentially resulting in storage-related loss of 20.7% to 40.7% of vitamin A (mean estimate for loss over 14 days: 30.7%). This estimate is similar to at least one study that reported 60–68% loss after a month of storage [27]. Vitamin A is further lost during cooking and one study assessed that 6% of vitamin A is lost after one frying [29]. Based on these estimates, we assumed that roughly 35% of vitamin A might be lost during storage and cooking. In order to account for losses, we multiplied daily individual oil consumed by 65% to estimate the amount of vitamin A potentially consumed from fortifiable edible oil by each person.

Finally, to understand the amount of vitamin A potentially available from fortified edible oil, we modeled fortification at three hypothetical levels—1) 15 μg/g RE, 2) 20 μg/g RE, and 3) 30 μg/g RE; derived from minimum, mid-point and upper limits of the oil fortification standard in Bangladesh. Using the calculations stated above in steps 1–3, potential post-fortification daily vitamin A intake in each sub-group was modeled for the entire population. Finally, Estimated Average Requirement (EAR)[30] for vitamin A was assigned to individuals based on their age and sex.

### Multidimensional poverty index

The MPI index scores range from 0 to 1, with a household classified as being in poverty if MPI≥0.33. Several surveys have used MPI indicators to identify households in poverty, which can help determine if the fortification interventions are reaching the most deprived populations [25, 31]. We adapted the core components of the MPI to create a comparable MPI index, which has been validated and used in fortification coverage surveys including the Bangladesh survey [17, 32].

### Data management and statistical analyses

Data were analyzed using STATA version 13.0 (StataCorp, College Station, TX). Oil purchase and poverty (in-poverty vs. not in-poverty defined using MPI score) were assessed at the household level, consistent with how the survey was administered. The survey also collected data on 1) household member’s age, which was used to compute the age ranges (< 12 months, 12–23 months, 24–59 months, 5–14 years, 15–19 years, 20–49 years and over 50 years,); 2) gender (female vs. male,); and 3) education (< 5 years or ≥5 years of education).

Our previous publication has addressed coverage, in detail [25]. Briefly, the following three summary statistics were calculated:

1. Contact coverage, defined as the proportion of all households that has coverage (i.e. the proportion of households using edible oil). This estimates overall program coverage.
2. Met need, defined as the proportion of households considered as vulnerable that has coverage (i.e. the proportion of vulnerable households that are using fortifiable edible oil). This estimates how well the program addresses vulnerability.
3. Coverage ratio, defined as the ratio of coverage in households considered vulnerable to the coverage in households that are not considered vulnerable. A coverage ratio of <1 indicates poor targeting (i.e. coverage is higher among the non-vulnerable than vulnerable population), whereas >1 signifies good targeting (i.e. coverage is higher among vulnerable population) [32].

The relationship between categorical variables was analyzed using the chi-squared test and continuous variables were analyzed using one-way ANOVA. Data were weighted for the relative proportion of the population in each stratum. Statistical weights were applied to all analyses to account for differences in population size of the strata and the probability of a household being selected from within each PSU. Two-sided statistical testing with a significance level of 0.05 was used.

## Results

Edible oil use was ubiquitous in Bangladesh; therefore contact coverage was estimated to be 100% at household level. Overall, 87.5% (n = 1,300) of the 1,512 households purchased fortifiable edible oil, irrespective of stratum and poverty (assessed based on MPI scores). **Table 1** describes the characteristics of households, categorized by the type of edible oil purchased (fortifiable vs. non-fortifiable). There were no significant differences in household size between the groups. A significantly greater percentage of urban households bought fortifiable edible oil (94.0%) when compared to households in rural other (88.1%) and rural low-performing strata (79.7%) (p value: 0.01). Households in poverty (MPI ≥ 0.33) were significantly less likely to purchase fortifiable edible oil (82.1%) when compared to those not in poverty (MPI <0.33) (91.4%) (p value: 0.001). Respondents in households that used fortifiable oil were more likely to have at least 5 years of education, than respondents in households that used non-fortifiable oil (p value: 0.008). Those households that primarily used non-fortifiable oil reported purchasing significantly less (29 ml/day) edible oil than the households that primarily used fortifiable oil (p value: <0.001).

**Table 1:** Household characteristics (weighted), stratified by the purchase of fortifiable edible oil in the household during the survey period (Jan–Apr 2015).

	Households purchasing fortifiable oil (n = 1,300)	Households purchasing non- fortifiable oil^a^ (n = 212)	p value
**Household characteristics,** mean (95% CI)			0.9
Household size	4.9 (4.7, 5.2)	4.9 (4.5, 5.3)	
**Household by strata,** % (95% CI)			0.01
Urban	94.0 (89.4, 96.7)	6.0 (3.3, 10.6)	
Rural low performing	79.6 (68.5, 87.6)	20.4 (12.4, 31.5)	
Rural other	88.1 (82.2, 92.2)	11.9 (7.8, 17.8)	
**Overall risk of poverty,** % (95% CI)			0.001
High MPI^b^	82.1 (75.0, 87.5)	17.9 (12.5, 25.0)	
Low MPI^b^	91.4 (87.9, 94.0)	8.6 (6.0, 12.1)	
**Education,** % (95% CI)			0.008
Respondent had ≥5 years of schooling	91.3 (87.1, 94.2)	8.7 (5.8, 12.9)	
Respondent had < 5 years of schooling	84.0 (77.8, 88.7)	16.0 (11.4, 22.2)	
**Edible oil amount (ml)** (mean, 95% CI)	116.0 (107.5, 124.4)	87.0 (76.1, 97.5)	<0.001

^a^ Non-fortifiable oil is defined as an edible oil that is not mandated to be fortified (e.g. mustard seed oil) or is home-made

^b^ MPI—Multidimensional poverty index; A household is categorized as being in poverty if MPI, a weighted index score which ranges from 0 to 1, is ≥0.33

**Table 2** summarizes the weighted met need coverage and coverage ratio of fortifiable edible oil for households at risk of poverty as well as rural households (both low-performing and other). The weighted met need coverage was 82.1% (95% CI: 75.0, 87.5) for households in poverty, 79.7% (95% CI: 68.5, 87.6) and 88.1% (95% CI: 82.2, 92.2) in rural low performing and rural other residences. The weighted coverage ratio for fortifiable edible oil use was 0.90 for households in poverty, 0.85 and 0.94 respectively for rural low performing and rural others.

**Table 2:** Summary statistics for coverage (met need and coverage ratio) of household edible oil by risk group.

Risk group	n	% Met need (95% CI)^a^	Coverage Ratio^b^
**Poverty**		** **	** **
MPI ≥ 0.33^**c**^	596	82.1 (75.0, 87.5)	0.90 (0.85, 0.93)
**Stratum**			
Rural low performing	504	79.7 (68.5, 87.6)	0.85 (0.77, 0.91)
Rural Other	504	88.1 (82.2, 92.2)	0.94 (0.92, 0.95)

^a^ Met need is defined as the proportion of households defined as at-risk (due to vulnerability to poverty or residence in rural low performing/ rural other areas) that has coverage

^b^ Coverage ratio is defined as ratio of coverage in at-risk households to the coverage in households that are not at risk

^c^ MPI—Multidimensional poverty index; A household is categorized as being in poverty if MPI, a weighted index score which ranges from 0 to 1, is ≥0.33

### Fortifiable edible oil at individual level

Overall, mean weighted per capita fortifiable edible oil consumption was 21.0 g/day (95% CI: 19.3, 22.8). Daily mean edible oil was assessed using the AME approach, which assumed that each person’s intake was proportional to sex- and age-specific caloric requirements and is presented by strata (S2 Table) and poverty (S3 Table). Mean fortifiable edible oil consumption in urban (24.6 g/day) and rural others (21.9 g/day) strata was significantly greater than in rural low performing (15.8 g/day). Similarly, individuals living in households not in poverty were estimated to consume significantly more fortifiable edible oil (23.6 g/day) than those in poverty (17.7 g/day).

### Modeled vitamin A intake from fortifiable edible oil

There were significant differences in the percent of daily vitamin A EAR that could potentially be met by fortification at 20 μg/g RE, a hypothetical mid-point of the oil fortification standard in Bangladesh, which ranges between 15–30 μg/g. Based on modeled estimates at 20 μg/g RE, individuals in rural low performing households were projected to meet 52.4% of daily vitamin A EAR from fortified oil, compared to 81.5% in urban (81.5%) and 72.8% in rural other households (Table 3). Similarly, vitamin A intake may likely vary with poverty, with fortified edible oil (20 μg/g RE) potentially meeting 59.2% (95% CI: 52.0, 66.4) of the vitamin A EAR for those living in households in poverty as opposed to 78.0% (95% CI: 73.4, 82.7) that are not in poverty (Table 4). Vitamin A EAR expected to be met through fortified edible oil was estimated to be significantly lower for females (68.5%; 95% CI: 62.7, 74.2) than males (71.2%, 95% CI: 65.2, 77.1) (p value– 0.001) (S4 and S5 Tables). Similar trends were observed when modeled at 15 and 30 μg/g RE, the minimum and upper limits of the Bangladesh oil fortification standards (Tables 3, 4, S4 and S5 Tables).

**Table 3:** Daily vitamin A EAR (%) expected to be met by consuming edible oil fortified at different levels of vitamin A (15 μg/g RE, 20 μg/g RE, 30 μg/g RE), stratified by age group and strata.

	Urban				Rural low performing				Rural Others			
Age range	N	15 μg/g RE	20 μg/g RE	30 μg/g RE	N	15 μg/g RE	20 μg/g RE	30 μg/g RE	N	15 μg/g RE	20 μg/g RE	30 μg/g RE
12–23 mo	44	33.9^a^ (30.5, 37.3)	45.2^a^ (40.6, 49.7)	67.8^a^ (61.0, 74.6)	52	26.0 (20.6, 31.4)	34.7 (27.5, 41.9)	52.1 (41.3, 62.9)	49	26.7 (23.1, 30.4)	35.6 (30.7, 40.5)	53.5 (46.1, 60.8)
24–59 mo	162	38.5^a^ (35.2, 41.8)	51.3^a^ (47.0, 55.5)	77.0^a^ (70.4, 83.5)	143	24.0 (17.5, 30.4)	32.0 (23.4, 40.6)	47.9 (35.0, 60.8)	164	34.9^b^ (30.6, 39.2)	46.5^b^ (40.8, 52.3)	69.8^b^ (61.1, 78.4)
5–14 y	443	52.1^a^ (47.4, 56.8)	69.5^a^ (63.63, 75.4)	104.2^a^ (94.8, 113.6)	536	31.8 (25.2, 38.5)	42.4 (33.6, 51.3)	63.7 (50.4, 76.9)	580	45.5^b^ (41.7, 49.3)	60.6^b^ (55.5, 65.7)	91.0^b^ (83.3, 98.6)
15–19 y	277	65.5^a^ (61.1, 69.9)	87.3^a^ (81.5, 93.2)	131.0^a^ (122.3, 139.8)	243	41.8 (37.3, 46.4)	55.8 (49.7, 61.9)	83.7 (74.5, 92.9)	245	60.8^b^ (54.1, 67.5)	81.1^b^ (72.1, 90.0)	121.6^b^ (108.2, 135.1)
20–49 y	1065	72.4^a^ (67.7, 77.1)	96.6^a^ (90.3, 102.9)	144.9^a^ (135.5, 154.3)	934	49.8 (43.1, 56.6)	66.5 (57.5, 75.4)	99.7 (86.2, 113.2)	1019	66.0^b^ (59.9, 72.2)	88.1^b^ (79.9, 96.2)	132.1^b^ (119.9, 144.3)
Over 50 y	312	56.4^a^ (51.8, 61.1)	75.3^a^ (69.1, 81.4)	112.9^a^ (103.6, 122.2)	381	38.4 (33.0, 43.8)	51.2 (44.0, 58.4)	76.8 (66.0, 87.5)	357	51.7^b^ (47.1, 56.3)	69.0^b^ (62.8, 75.1)	103.4^b^ (94.2, 112.7)

^a^—Significant difference in percentage vitamin A EAR (weighted) met by consuming vitamin A fortified edible oil when comparing urban and rural low performing household members (p <0.05)

^b^—Significant difference in percentage vitamin A EAR (weighted) met by consuming vitamin A fortified edible oil when comparing rural other and rural low performing household members (p <0.05)

When a superscript is not included in the table, it means that there is no significant difference between rural low performing and urban or rural others

**Table 4:** Daily vitamin A EAR (%) expected to be met by consuming edible oil fortified at different levels of vitamin A (15 μg/g RE, 20 μg/g RE, 30 μg/g RE), stratified poverty level and age range.

	Poverty (MPI ≥ 0.33) ^b^				Not in poverty (MPI (<0.33) ^b^				Overall			
Age range	N	15 μg/g RE	20 μg/g RE	30 μg/g RE	N	15 μg/g RE	20 μg/g RE	30 μg/g RE	N	15 μg/g RE	20 μg/g RE	30 μg/g RE
12–23 mo	55	22.9 (19.5, 26.3)	30.6 (26.0, 35.1)	45.8 (39.0, 52.6)	90	31.5^a^ (27.4, 34.7)	42.1^a^ (36.5, 46.2)	63.1^a^ (56.1, 70.1)	145	28.3 (25.9, 30.7)	38.0 (34.5, 41.0)	56.6 (51.8, 61.5)
24–59 mo	177	27.7 (22.7, 32.8)	37.0 (30.2, 43.7)	55.5 (45.3, 65.6)	283	36.7^a^ (33.8, 40.1)	48.9^a^ (45.1, 53.5)	73.4^a^ (67.2, 79.6)	469	33.3 (30.1, 36.6)	44.4 (40.1, 48.8)	66.7 (60.2, 73.1)
5–14 y	855	37.9 (32.0, 43.7)	50.5 (42.7, 58.3)	75.7 (65.1, 87.4)	678	49.9^a^ (47.1, 53.1)	66.5^a^ (62.8, 70.8)	99.8^a^ (93.5, 106.2)	1559	42.8 (38.1, 47.5)	57.0 (50.8, 63.3)	85.6 (76.1, 95.0)
15–19 y	236	48.8 (42.5, 55.1)	65.5 (56.6, 73.5)	97.6 (84.9, 110.2)	516	62.1^a^ (57.2, 67.1)	82.9^a^ (76.5, 90.5)	124.3^a^ (114.5, 134.1)	765	57.3 (51.7, 62.9)	76.4 (69.0, 83.8)	114.6 (103.5, 125.7)
20–49 y	1065	55.3 (50.0, 60.5)	73.7 (66.7, 80.7)	110.5 (100.1, 121.0)	1881	69.6^a^ (65.3, 73.9)	92.8^a^ (87.3, 99.1)	139.2^a^ (130.7, 147.7)	3018	63.9 (59.3, 68.5)	85.2 (79.1, 91.4)	127.8 (118.6, 137.1)
Over 50 y	315	44.2 (39.0, 49.4)	58.9 (52.0, 65.8)	88.4 (78.0, 98.8)	679	52.5^a^ (48.9, 56.1)	70.0^a^ (66.2, 75.7)	105.1^a^ (97.9, 112.2)	1050	49.7 (46.3, 53.1)	66.3 (61.8, 70.8)	99.4 (92.6, 106.1)

^a^—Significant difference in percentage vitamin EAR (weighted) met by consuming vitamin A fortified edible oil between those household members, categorized by poverty status(p <0.05)

^b^–MPI—Multidimensional poverty index; A household is categorized as being in poverty if MPI, a weighted index score which ranges from 0 to 1, is ≥0.33

No superscript indicates that that there is no significant difference between members from households with or without poverty

**Fig 1:**
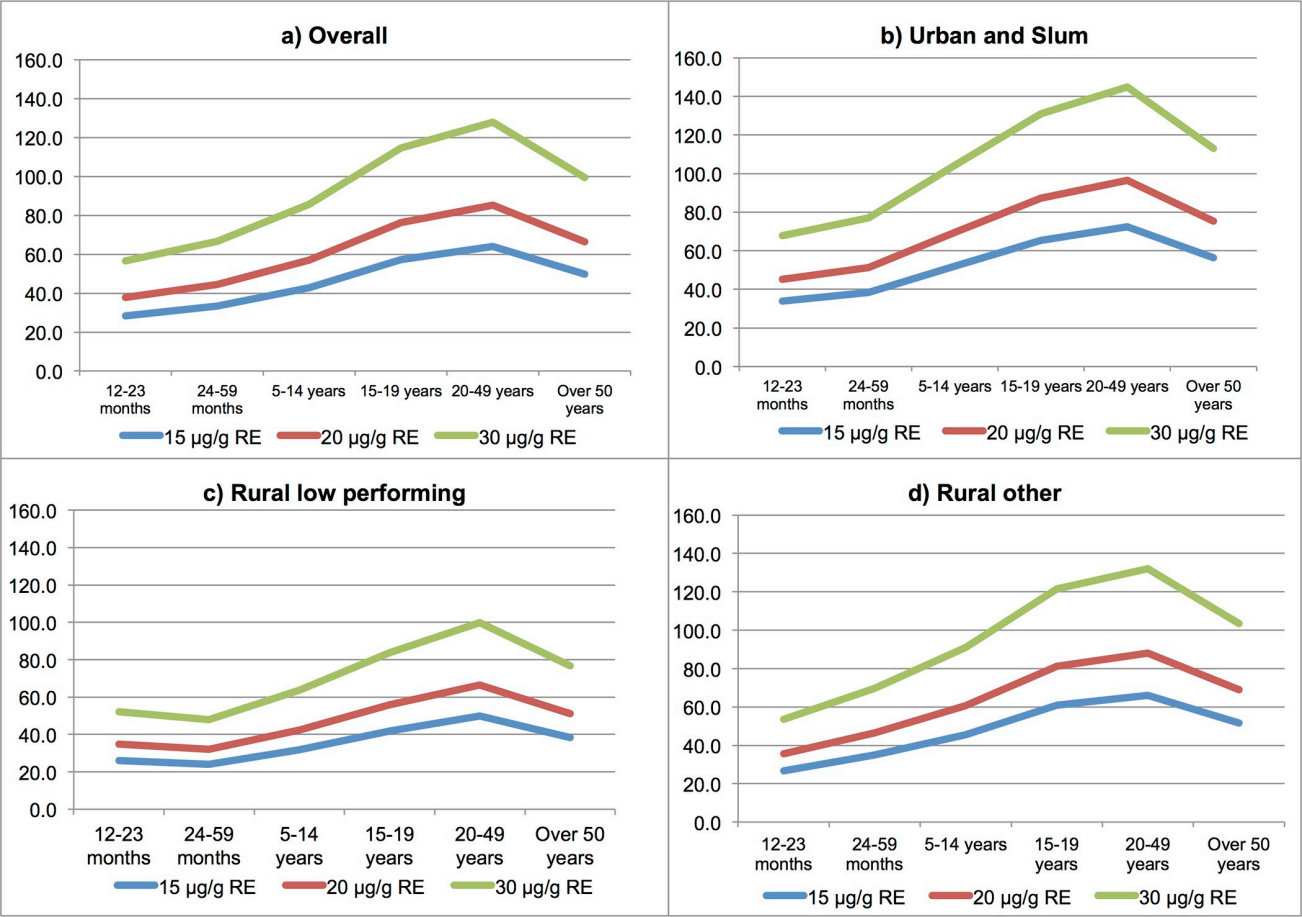
Daily vitamin A EAR (%) expected to be met by consuming edible oil fortified at different levels of vitamin A (15 μg/g RE, 20 μg/g RE, 30 μg/g RE), presented by strata and age groups.

When stratified by age, adolescents (15–19 years) and adults (20–49 years) would potentially receive more than three-quarters of their daily EAR for vitamin A from oil fortified at 20 μg/g RE (76.4%; 95% CI: 69.0, 83.8 and 85.2%; 95% CI: 79.1, 91.4, respectively) (Fig 1). Children (12–23 months and 24–59 months) would be expected to meet about half or less than half of their EAR for vitamin A through consumption of oil fortified at 20 μg/g RE (37.8%; 95% CI: 34.5, 41.0 and 44.4%; 95% CI: 40.1, 48.8) and this trend was consistent across strata.

## Discussion

Our study found that commercially produced vitamin A fortifiable edible oil (soybean and palm oil) is a commodity commonly consumed by the majority of Bangladeshi population and its consistent fortification according to national standards would partially meet a significant percentage of the daily vitamin A EAR requirements for most of the population. Thus, soybean and palm oil are appropriate vehicles for mass fortification in order to prevent or mitigate vitamin A deficiency. Although the coverage ratio is less than 1.0, about 80% of households in poverty can still be reached using this vehicle. A motivation for our analysis was to establish a baseline for met need and coverage ratio from which to monitor the impact of the national legislation for vitamin A fortification of these oils. The fact that our results show that access to fortifiable edible oil is widespread provides evidence from which to advocate that fortification should be mandatory, well regulated and enforced. Subsequent sentinel monitoring could be conducted on selected populations for tracking trends, progress and assessing whether the program is effective at the national and sub-national levels [33, 34].

### Equity of vitamin A fortification of edible oil

Despite the potential for broad reach of fortifiable edible oil, many studies have shown that the access to fortified foods is not equitable and the coverage is sometimes lowest among those that need the intervention the most [35, 36]. Along those lines, our results show that households in poverty have lower coverage of fortifiable oil and even when they have access to the product, they consume it in lower quantities. There are various reasons why inequities in access and use persist; however, these are seldom well-researched and reported [35]. Research that does exist in Bangladesh, suggests that fortifiable oil may be less available in poorer areas and, when available, may be prohibitively expensive for households in poverty [8]. It is also possible that these deprived households may have opted for home produced oil or oil manufactured by local small-scale industries, both of which are generally not fortified [3]. Low consumption of fortifiable oil may also be reflective of physical environment, cultural and social preferences [37].

Inequities in access to, and consumption of fortifiable staple products includes geographic inequities (urban, rural divide) and differences in age groups [38]. Studies in many low resource settings have shown that per capita edible oil and fat consumption are higher in urban than in rural households [39–41]. For instance, estimates from the early 1990s in Bangladesh suggest that urban populations consumed eight times more dietary fat when compared to rural populations [41]. Although the urban rural gap has been narrowing in the past few decades, there still exists some divide based on urban rural residence type[42], as observed in our data. Our analysis based on modeled vitamin A intake data suggests that some sub-groups might not meet adequate coverage because of their life stage and this may be even more pronounced for those living in rural areas and/or in poverty. Infants and pre-school aged children despite being most vulnerable to micronutrient deficiency [5], also potentially receive lower amounts of vitamin A from fortifiable edible oil. In order to meet the complete dietary needs of these sub-groups, multiple intervention strategies including alternative fortification vehicles or targeted feeding programs should be considered.

An important concern with edible oil fortification is the potential for excess consumption of vitamin A, at least for some sections of the population. Fortifying edible oil at the highest levels (30 μg/g RE) may provide a high percentage of EAR for some sub-groups (e.g. adolescents and adults). However, it is unlikely that individuals will exceed tolerable upper intake levels for vitamin A (600 μg/d for children <3 years of age and 3000 μg/d for adults) from fortified oil alone or in combination with vitamin A from multiple food vehicles [16, 43].

### Importance of implementation of mandatory fortification policy

Mandatory national fortification, although important, does not guarantee increased coverage of fortified products and associated nutritional benefits. For example, despite laws on mandatory vitamin A fortification, in Pakistan, only a few companies implement fortification, resulting in variable amounts of vitamin A in fortified oil and ghee brands [5]. Also, industry self-reported results in low- and middle-income countries in Africa and Asia suggest that only 45% of product samples are adequately fortified [44]. Thus, in order to maximize the impact of oil fortification in Bangladesh, it is important that adequate regulatory monitoring and quality assurance mechanisms should be put in place, with specific definitions of responsibilities for government and industry in order to achieve and sustain effective edible oil fortification [38, 45]. In addition, well-trained cadre of food inspectors, quality laboratories, and appropriate data capture mechanisms and most importantly, strong and committed government leadership is required to ensure program’s success [44].

### Limitations and strengths

This study has some limitations to report. First, no distinction was made between branded and unbranded oil. Data suggests that loosely packaged oil (likely unbranded) makeup 75% of the edible oil market share and in this modeling exercise it was assumed that both loosely packaged and bottled soybean and palm oils will comply to fortification [8]. Second, possible vitamin A variable losses during storage at the retail level were not accounted for and this could have resulted in measurement error than a systematic bias [27]. Third, the households consuming non-fortifiable edible oil could still be purchasing some amounts of fortifiable oil (soybean and palm oil), however, this study only captured main oil purchased, a majority of the times. Fourth, data collected for this survey is self-reported household level edible oil purchase data. While respondents may have accurately recalled whether they purchased edible oil or not, the quantity purchased may have been less precise. Fifth, edible oil estimates were calculated based on HCES approach in which assumptions were made about the intra-household food distributions. Further, the validity of AME approach is still being established. Thus, this approach may be less accurate than other methods such as 24-hour recall; however, evidence suggests that it is sensitive to distinguish consumption patterns among population strata [22]. Sixth, vitamin A intake calculated in this study is based on statistical modeling with notable assumptions and a lack of accounting for measurement error in the models. Seventh, although our study sheds light on potential vitamin A intake available through implementing oil fortification, it is not designed to assess the extent of vitamin A deficiency that could be mitigated through this fortification effort. Finally, this study estimated potential vitamin A intake from oil fortification and the amount of vitamin A from supplementation, although important, was not accounted for. Thus, lack of coverage data for all vitamin A interventions in Bangladesh may limit the understanding of the potential impact of nation-wide oil fortification efforts.

Despite these limitations, our study has strengths to highlight. The data for this study is from a large, nationally representative, stratified survey, which allowed for informative sub-group analyses to determine the likely impact of vitamin A fortified oil intervention among various socio-demographic groups in Bangladesh. An added advantage is that the study used standardized and validated indicators that were used to assess the need and risk. The results presented here provide an indirect but useful method for estimating the potential contribution to dietary vitamin A intake from implementation of edible oil fortification in Bangladesh. Thus, our findings have direct and important programmatic implications and can provide additional evidence for setting policy in Bangladesh.

In conclusion, fortification of edible oil with vitamin A has been shown to be a simple and cost-effective approach for addressing vitamin A status, which has the potential to reach large segments of the population in Bangladesh. It will be important for the Bangladesh government to monitor equity of access to the fortified product and, where needed, to implement strategies to increase access to the product among the more vulnerable groups identified by this study, and complement with alternative interventions, as needed. In addition, mechanisms need to be established to ensure product quality and compliance.

## Supporting information

S1 Table Household questionnaire. (PDF)

S2 Table Estimated weighted mean oil (g/d) consumption by age group, gender and strata. ^a^—Significant difference in weighted mean oil intake between urban and rural low performing household members (p <0.05).
^b^—Significant difference in weighted mean oil intake between rural other and rural low performing household members (p <0.05).
When a superscript is not included in the table means that there was no significant difference between rural low performing and urban or rural others.
(DOCX)

S3 Table Estimated weighted mean oil consumption (g/d) by age group, gender and poverty level. ^a^—Significant difference in weighted mean oil intake between non-deprived household members with low MPI (MPI <0.33) and deprived household members vulnerable to poverty (MPI ≥ 0.33) (p <0.05).
*—Total may not add up because of missing data.
When a superscript is not included in the table means that there was no significant difference between non-deprived household members with low MPI and deprived household members with high MPI (MPI ≥ 0.33).
(DOCX)

S4 Table Daily vitamin A EAR (%) expected to be met by consuming edible oil fortified at different levels of vitamin A (15 μg/g RE, 20 μg/g RE, 30 μg/g RE), stratified by strata, age group and gender. M–Male; F–Female.
^a^—Significant difference in percentage vitamin A EAR (weighted) met by consuming vitamin A fortified edible oil when comparing urban and rural low performing household members (p <0.05).
^b^—Significant difference in percentage vitamin A EAR (weighted) met by consuming vitamin A fortified edible oil when comparing rural other and rural low performing household members (p <0.05).
^c^–Weighted mean. When a superscript is not included in the table, it means that there is no significant difference between rural low performing and urban or rural others
(DOCX)

S5 Table Daily vitamin A EAR (%) expected to be met by consuming edible oil fortified at different levels of vitamin A (15 μg/g RE, 20 μg/g RE, 30 μg/g RE), stratified by age group, gender and poverty level. ^a^—Significant difference in percentage vitamin EAR (weighted) met by consuming vitamin A fortified edible oil between those household members, categorized by poverty status, based on households with high MPI (in poverty) and low MPI (not in poverty) (MPI <0.33) (p <0.05).
^b^–Weighted mean.
No superscript indicates that that there is no significant difference between household members between low MPI (not in poverty) and high MPI (in poverty).
(DOCX)

## Abbreviations

AME: Adult Male Equivalent
AME_Person_: Adult Male Equivalent of an individual
AME_HH_: Adult Male Equivalent of the household
GAIN: Global Alliance for Improved Nutrition
EAR: Estimated Average Requirement
HCES: Household Consumption and Expenditure Survey
icddr: b: International Center for Diarrhoeal Disease Research, Bangladesh
MICS: Multiple Indicator Cluster Survey
MPI: Multidimensional Poverty Indicator
PSU: Primary Sampling Units
RE: Retinol Equivalents
