# Supplementary material for: Household coverage of vitamin A fortification of edible oil in Bangladesh
Source: PLoS One. 2019 Apr 3;14(4):e0212257. doi: 10.1371/journal.pone.0212257 (PMC6447147; doi:10.1371/journal.pone.0212257)
Supplement: S1 Table — (PDF) [file pone.0212257.s001.pdf]

Household QUESTIONNAIRE

Date : |__|__|.|__|__|.|__|__||__|__|

PSU ID: PSU identifier: |__|__|__|

PSU NAME: Name of village / town / quarter / block:--

HH ID: Household identifier: |__|__|

IN ID: Interviewer identifier:” |__|__|__|

**CONSENT**	*Was household consent obtained?*	*01=Yes*<br>*02 = Refusal*<br>*03= Occupants not present*	[__]

Request an interview with the following person (in order of preference)<br>*RC1: The person mainly responsible for cooking in the household (minimum age limit 15 years).*<br>*RC2: A woman of reproductive age (15-49 years) present at the time of the visit.*<br>*RC3: Another adult household member present at the time of the visit (minimum age limit 18 years).*

**Category of the respondent (arranged in order of preference)**<br>*Select the respondent category with an “√”*		
**RC1** ☐	**RC2** ☐	**RC3** ☐

IN: (Interviewee Sex, Age and Education)

	QUESTION	CODING CATEGORIES	CODE
**IN1**	Name<br>--	Mobile Number<br>☐☐☐☐☐☐☐☐☐☐☐<br>Name:--------------------------------------<br>Relation of phone owner with respondent	
**IN2**	Sex (M/F)	Male...01<br>Female..02	☐☐

**IN3**	Age (years)	Complete years	
**IN4**	Have you ever attended school or college?	Yes……………………………………………..01<br>No………………………………………………02	02⇨HH1
**IN5**	How many years of education did you have?	<5 years...01<br>5-10 years..02<br>SSC...03<br>HSC...04<br>Graduation...05<br>Masters...06<br>Others...07	
**IN6**	Currently attending school or college?	Yes……………………………………………….01<br>No………………………………………………..02	

Household Members Information

For interviewer use only
Household member definition:
One person living alone, or a group of people (not necessarily related) living at the same address with common housekeeping (sharing either a living room or sitting room, or at least one meal a day)

	QUESTION	CODING CATEGORIES	CODE
**HH1**	How many people live in this household?	-------------------Person	
		Go to SBH1 if HH1 =1	
**HH2**	How many household members are male?	-------------------Person	
		If there is no male member in this HH go to HH3	
How old are the <HH2> males in the house?			
**HH2a**	0-11 months (<1 year)	-------------------Person	
**HH2b**	12-23 months (1-< 2 years)	-------------------Person	
**HH2c**	24-59 months (2-<5 years)	-------------------Person	
**HH2d**	5 to 14 years	-------------------Person	
**HH2e**	15 to 19 years	-------------------Person	
**HH2f**	20 to 49 years	-------------------Person	
**HH2g**	50 years or more	-------------------Person	
**HH3**	Confirm that there are (HH1-HH2) females in the household	------------------- Person	

	QUESTION	CODING CATEGORIES	CODE
How old are the (HH3) females in the house?			
**HH3a**	0-11 months (<1 year)	-------------------Person	
**HH3b**	12-23 months (1-< 2 years)	------------------- Person	

**HH3c**	24-59 months (2-<5 years)	------------------- Person	
**HH3d**	5 to 14 years	------------------- Person	
**HH3e**	15 to 19 years	------------------- Person	
**HH3f**	20 to 49 years	------------------- Person	
**HH3g**	50 years or more	------------------- Person	

SBH: Short Birth History-MPI mortality

Follow this module if there is any woman of reproductive age in this household (check HH3e-HH3f)
The purpose of the short birth history (SBH) component is to determine the number of live births to CURRENT members of the household and establish where these children are now.

	QUESTION	CODING CATEGORIES	CODE
**SBH1:**	MZ 5 eQ‡ii g‡a¨ GB Lvbvq †Kvb Rb¥ n‡q‡Q?	Yes……………………………………………......01<br><br>No…………………………………………….........02	02⇨ED1

For interviewer use only
Only consider the birth of a child, if it has occurred to a ***current*** *member of the household. (i.e. if there was a child birth in this HH, but both of the parents now live outside this HH, then do not consider it a "recent birth in the HH". However, if a child was born to parents, who (both or either one) currently live in the HH, even though they did not live in the HH when the child was born, consider it a "recent birth in the HH".*
Tell the respondent:

- *I will be asking about recent births in the household.*
- *I am interested in all children that were born alive.*

Ask the following questions:

SBH2:	How many live births have there been in the household in the last 5 years?	Rb☐

Complete this table for live births to current members of the household in the last 5 years.			
No	Rb¥ ZvwiL		Where are they now?
	Name of Month	Year	*1= Alive 2=dead*
**1**			|__|
**2**			|__|
**3**			|__|
**4**			|__|
**5**			|__|
**6**			|__|

	QUESTION	CODING CATEGORIES	CODE
FOR INTERVIEWER USE ONLY *If any response in the table above shows that a child has died then record a* ***Yes*** *here, otherwise record* ***No***			
**D1:**	*A recently born child had died?*	Yes……………………………………………….01<br>No……………………………………………….02	|__|__|

ED: Schooling

	QUESTION	CODING CATEGORIES	CODE
**ED1**	Does any other household member have 5 or more years of schooling?	Yes……………………………………………….01<br>No……………………………………………….02	|__|__|

	If there is no child in the HH aged between 5 and 14 years (check HH2d and HH3d) ***skip to M1***		
**ED2**	Is there any child in the HH aged between 5 & 14 years who does <u>not</u> attend school?	Yes……………………………………………..........01<br>No……………………………………….….….......02	☐☐

M: MPI assets data

In this section I will ask about the assets of the household and questions about where you get your water and what kind of latrine do you use.

	QUESTION	CODING CATEGORIES	CODE
M1:	Does your household have electricity?	Yes……………………………………………01<br>No……………………………………………02	☐☐
	QUESTION	CODING CATEGORIES	CODE
M2:	What fuels does your household use for cooking?<br>*Multiple responses allowed.*<br>*Allow respondent to respond freely, and then prompt "Anything else?" Do not specify the fuels from the list.*		
M2a:	Electricity<br>If the response is yes but the response to M1 was no then check M1 again with the respondent.	Yes……………………………………………01<br>No……………………………………………02	☐☐
M2b:	Gas/LP Gas	Yes……………………………………………01<br>No……………………………………………02	☐☐
M2c:	Kerosene	Yes……………………………………………01<br>No……………………………………………02	☐☐
M2d:	Coal / lignite	Yes……………………………………………01<br>No……………………………………………02	☐☐
M2e:	Charcoal	Yes……………………………………………01<br>No……………………………………………02	☐☐
M2f:	Wood	Yes……………………………………………01<br>No……………………………………………02	☐☐
M2g:	Straw / shrubs / grass / crop residue	Yes……………………………………………01<br>No……………………………………………02	☐☐
M2h:	Animal dung	Yes……………………………………………01<br>No……………………………………………02	☐☐
M2i:	Biogas	Yes……………………………………………01<br>No……………………………………………02	☐☐
M2j:	Others (Please specify)	Yes……………………………………………01<br>No……………………………………………02	☐☐

**M3 :**	What is the main material of the floor of the dwelling?<br>*Record the main material of the floor of the dwelling from observation. If not possible then ask respondent.*		
M3a:	Dirt / earth / sand / dung / mixed	Yes……………………………………………..01<br>No……………………………………………..02	☐☐
M3b:	Wood/Bamboo	Yes……………………………………………..01<br>No……………………………………………..02	☐☐
M3c:	Brick-cement/tiles	Yes……………………………………………..01<br>No……………………………………………..02	☐☐
**M4:**	Does your household have...?<br>*Read and prompt for each item. Record <u>all</u> items owned by household*		
M4a:	A radio?	Yes……………………………………………..01<br>No……………………………………………..02	☐☐
M4b:	A television?	Yes……………………………………………..01<br>No……………………………………………..02	☐☐
M4c:	A Refreegerator television?	Yes……………………………………………..01<br>No……………………………………………..02	☐☐
M4d:	A cassette/CD player?	Yes……………………………………………..01<br>No……………………………………………..02	☐☐
M4e:	A mobile or non-mobile telephone?	Yes……………………………………………..01<br>No……………………………………………..02	☐☐

M4f:	A motorcycle/ scooter/Auto-rickshaw (CNG)?	Yes……………………………………………..01<br>No………………………………………………02	
M4g:	A rickshaw/van/bicycle?	Yes……………………………………………..01<br>No………………………………………………02	
M4h:	A motor rickshaw/ Easy-bike?	Yes……………………………………………..01<br>No………………………………………………02	
M4i:	Car/ Truck/Tractor?	Yes……………………………………………..01<br>No………………………………………………02	
M4k:	A boat with motor?	Yes……………………………………………..01<br>No………………………………………………02	
M4l:	A cow/goat/hen/duck?	Yes……………………………………………..01<br>No………………………………………………02	
M4m:	A computer/laptop?	Yes……………………………………………..01<br>No………………………………………………02	

W: Water, Sanitation and Hygiene (WASH)

	QUESTION	CODING CATEGORIES	CODE
**W1 :**	What is the main source of your drinking water? *(enter **one** code only)*		
W1a:	Piped water into dwelling	01	
W1b:	Piped water into yard/plot/compound	02	
W1c:	Public tap/standpipe	03	
W1d:	Tube-well/borehole	04	
W1e:	Protected dug well/spring	05	
W1f:	Rainwater collection system	06	
W1g:	Bottled water/20L container	07	
W1h:	Unprotected dug well/spring	08	
W1i:	Cart wirth small tank or drum	09	
W1j:	Tanker truck	10	
W1K:	Surface water (river/dam/lake/pond/stream/canal/irrigation)	11	
W1l:	Others (please specify) ……………………………………………………….	77	

**W2 :**	What do you usually do to the water to make it safer to drink?<br>*(Do not prompt. Enter one code only)*		
W2a:	Boil	01	☐☐
W2b:	Add bleach/chlorine tablet/Potash Alum	02	
W2c:	Water filter (Ceramic/gravel/sand)	03	
W2d:	Solar disinfectant	04	
W2e:	Strain it through a cloth only	05	
W2f:	Let it stand and settle only	06	
W2g:	Nothing	07	
W2h	Others (Please specify)<br>..	77	
W2i	Don't know	99	
**W3 :**	What kind of toilet facility do members of your household usually use?<br>*In case the respondent is unable to tell the type of toilet, ask for permission to observe it if possible (Enter one code only)*		
Flush/ Pour flush System, that flushes to:			☐☐
W3a:	Piped sewer system	01	
W3b:	Septic tank	02	
W3c:	Pit latrine with water seal	03	
W3d:	Somewhere else	04	
W3e:	Don't know where	05	
Pit latrine (without water seal)			
W3f:	Ventilated Improved Pit	06	
W3g:	Pit latrine with slab without water-seal	07	
W3h:	Pit latrine without slab and water-seal	08	
W3i:	Composting latrine	09	
W3j:	Bucket latrine	10	
W3k:	Hanging Latrine	11	
W3l:	No facilities/bush/field	12	
W3m:	Others (please specify)<br>..	77	

			⇨W5
**W4:**	Do you share this facility with other households?	Yes……………………..…..01<br>No…………………….......02	☐☐
If there is a child under 2 years in the household, (Check answer to Question HH2a-HH2c and HH3a-HH3c) ask W5. If not GOTO W6			
**W5 :**	The **last time** the child under 2 years old passed stools, what was done to dispose of the stools? (*Do not prompt)*<br>*(Enter **one** code only)*		
W5a:	Child used toilet/latrine	01	☐☐
W5b:	Put into toilet/latrine	02	
W5c:	Put into drain/ditch	03	
W5d:	Thrown into garbage	04	
W5e:	Nothing/left it open	05	
W5f:	Don't know	99	
W6:	Please tell me all of the occasions when it is important for you to wash hands with soap<br>*Do not prompt, Probe 'Anything Else?' Enter all codes that apply*		
W6a:	Before Eating	Yes……………………..…..01<br>No…………………….......02	☐☐
W6b:	Before feeding a child	Yes……………………..…..01<br>No…………………….......02	☐☐
W6c:	Before cooking/preparing food	Yes……………………..…..01<br>No…………………….......02	☐☐
W6d:	After defecation/urination	Yes……………………..…..01<br>No…………………….......02	☐☐
W6e:	After cleaning a child that has defecated/changing nappies/washing diaper	Yes……………………..…..01<br>No…………………….......02	☐☐

W6f:	Others (please specify)<br>...	Yes…………………………..01<br>No…………………………..02	☐☐
W6g:	Don't know/No answers given	Yes…………………………..01<br>No…………………………..02	☐☐

H: Household hunger scale (food security)

The following questions are to determine whether your household or you have experienced any shortage of food in the past month.

For interviewer use only

In general:

00 = never
01 = Rarely = (1 to 2 times)
02 = Sometimes = (3 to 10 times)
03 = Often = (more than 10 times)

You may need to probe beyond the initial answer to determine which of the above categories to record.

H1: How many times in the **last month** was there ever no food to eat of any kind in your house because of lack of resources to get food? |__|__|
(if none put '00')

H2: How many times in the **last month** did you personally go to sleep at night hungry because there was not enough food? |__|__|
(if none put '00')

H3: How many times in the **last month** did you personally go a whole day and night without eating anything at all because there was not enough food? |__|__| *(if none put '00')*

NUT: Nutrition data Women of Reproductive Age (WRA)

BOX 1 - FOR INTERVIEWER AND SUPERVISOR USE ONLY

WRA: *Number of women 15-49 years of age living in this household?* *|__| If 0 GOTO AP1*
(Calculate from ***HH3e*** *and* ***HH3f****)*

Is the interviewee a woman of reproductive age? *Yes = 1, No= 2 |__|*

IF THE INTERVIEWEE IS A WRA THEN REQUEST WHETHER YOU MAY TAKE A MEASUREMENT OF THEIR LEFT UPPER ARM CIRCUMFERENCE TO PROVIDE SOME ASSESSMENT OF THEIR NUTRITIONAL STATUS

If the interviewee is not a wra, check box 1 to see if any wra in the household, ask if they are present and request whether you may take a measurement of their left upper arm circumference to provide some assessment of their nutritional status

---

NUT: Age of selected woman in years *(check this is 15-49 years)* |__|__|
Enter 77 if no WRA consents to the measurement

MUAC on the left arm (1st time) |__|__|.|__| cm

MUAC on the left arm (2nd time) |__|__|.|__| cm

Enter:

77.7 if MUAC too big (MUAC tape does not fit)
Record in what condition the MUAC was measured (place tick)

Bare skin	|__|
Clothing (thin)	|__|
Clothing (thick)	|__|
Bangles	|__|

IF MUAC < 21.0 CM → REFER TO A HEALTH FACILITY.

IODINE AND IODISED SALT-BANGLADESH

**AP: Awareness and practices related to IDD and iodized salt**			
	QUESTION	CODING CATEGORIES	CODE

**AP1.**	Have you ever heard of iodine as part of nutrient?	Yes..............................01<br>No...............................02	☐☐<br>02 ⇨AP5
**AP2.**	Do you know what problems one might have if s/he has iodine deficiency?<br>*(Multiple responses possible)*<br>*(Do not read responses, respond for **<u>All</u>** that are mentioned then return and complete "2" when the respondent confirms that they have no more responses.)*		
**AP2.a**	Development of Goitre	Yes..............................01<br>No...............................02	☐☐
**AP2.b**	Chance of complication during delivery/increases chance of miscarriage and stillbirth	Yes..............................01<br>No...............................02	☐☐
**AP2.c**	Hampers growth in neonates/children	Yes..............................01<br>No...............................02	☐☐
**AP2.d**	Reduces child intelligence/ Intellectual develpoment	Yes..............................01<br>No...............................02	☐☐
**AP2.e**	Hampers health in adolescents/adults	Yes..............................01<br>No...............................02	☐☐
**AP2.f**	Hampers normal brain development	Yes..............................01<br>No...............................02	☐☐
**AP2.g**	Development of Cretinism	Yes..............................01<br>No...............................02	☐☐
**AP2.h**	Others (please specify)	Yes..............................01<br>No...............................02	☐☐
**AP2.i**	Don't know any answers	Yes..............................01<br>No...............................02	☐☐
**AP3.**	Do you know of any method to prevent iodine deficiency?	Yes..............................01<br>No...............................02	☐☐

			02 ⇨AP5
**AP4.**	Please state the <u>most important</u> way to prevent iodine deficiency	Use iodised salt....................01<br>Take iodine supplements..................02<br>Eat seafood/seaweed ....03<br>Other (Please specify) ....077<br>Don't Know......................................99	
**AP5.**	Have you heard about iodized salt?	Yes…..........................01<br>No…….........................02	02 ⇨AP12
**AP6:**	Where have you heard about iodized salt?<br>*(Multiple responses possible. Do not prompt, probe "Anything else?" Respond for All that are mentioned then return and complete "2" when the respondent confirms that they have no more responses)*		
**AP6.a**	Television	Yes…..........................01<br>No…….........................02	
**AP6.b**	Radio	Yes…..........................01<br>No…….........................02	
**AP6.c**	Newspaper	Yes…..........................01<br>No…….........................02	
**AP6.d**	Poster/Leaflet	Yes…..........................01<br>No…….........................02	
**AP6.e**	Doctor/ Health worker/<br>NGO worker	Yes…..........................01<br>No…….........................02	
**AP6.f**	Relatives	Yes…..........................01<br>No…….........................02	
**AP6.g**	Friend/Neighbour	Yes…..........................01<br>No…….........................02	

**AP6.h**	teacher/School	Yes……………………….01<br>No………………………..02	☐☐
**AP6.i**	Shopkeeper	Yes……………………….01<br>No………………………..02	☐☐
**AP6.j**	Others ()	Yes……………………….01<br>No………………………..02	☐☐
**AP7**	Do you think, one can prevent iodine deficiency only by consuming iodized salt?	Yes……………………….01<br>No………………………..02<br><br>It depends on the severity of deficiency .... 03<br>Don't know.................................99	☐☐<br><br>01⇨ AP9<br>99⇨AP9
**AP8**	If no or "it depends", then what is the most important reason behind this?<br>*(Do not prompt)*	It is not possible to get adequate iodne from salt..............................01<br>It is harmful for health to consume excess table salt…………………………..02<br>Others (Please specify)....................03	☐☐
**AP9**	Do you use iodized salt for cooking in your household?	Yes……………………….01<br>No………………………..02<br>Don't know.................................99	02⇨ AP11<br>99⇨AP12

**AP10**	If yes, what is the most important cause for doing this?<br><br>*(Do not read responses. Select one option only. If more than one response is given please probe to find the most important reason)*	Consuming iodized salt is good for health ………….01<br>No other type of salt is available……..02<br>Advised by doctor……………………………..03<br>Suggested by shopkeeper ………………..04<br>Advised by relatives/friend/neighbour ………05<br>Inspired by TV/radio/Newspaper.........06<br>Others (please specify) 77	☐☐
	If Ans to **AP9** is 01, then skip **AP11**		
**AP11**	If not, what is the most important cause for not doing so?<br><br>*(Do not read responses. Select one option only. If more than one response is given please probe to find the most important reason)*	Not readily available.........................01<br>More expensive than non-iodised.....................02<br>I/household members don't like the taste .....03<br>Not always available in small quantities ....04<br>Others (please specify)..............077<br>Don't Know......................................99	☐☐
**AP12**	Do you or any of your household members use table salt?	Yes……………………….01<br>No…………………………02	☐☐<br>02⇨ AP15

**AP13**	If yes, please confirm which members used table salt in the past week?	Age	Number of household members *(Record from HH2 and HH3)*	Number of members using table salt
		<5 years		
		5-14 years		
		15-19 years		
		20-49 years		

		50 years or more		
**AP14**	Do you use idoized salt as table salt in your household?	Yes.............................01<br>No..............................02<br>Don't know................99		☐☐
**AP15**	Is there any member in your household who has delayed mental development?	Yes.............................01<br>No..............................02		☐☐<br>02⇨ AP17
**AP16**	How many members are there and please mention their age	Serial no	Age	
		1		
		2		
		3		
**AP17**	Is there any member in your household who has goitre?	Yes.............................01<br>No..............................02		☐<br>02⇨ AP19
**AP18**	How many members are there and please mention their age	Serial no	Age	
**AP19**	Do you know that there is a "salt law"?	Yes.............................01<br>No..............................02<br>Don't know 99		☐☐<br>02 ⇨ AP21<br>99 ⇨ AP21
**AP20**	Do you know whether there is any punishment for violating this law for retailers/manufacturers?	Yes...01<br>No..02<br>Don't know..................................99		☐☐
**AP21**	Do you know whether salt can be tested for iodine?	Yes.............................01<br>No..............................02		02 ⇨ AP26
**AP22**	If yes, please state the methods of testing salt for iodine	With home ingredients<br>.............................01<br>With testing kit/any chemicals<br>.................................02<br>Both..03<br>Don't know....................................99		☐☐<br>02 ⇨ AP24<br>99 ⇨ AP26<br>☐☐

**AP23**	What are the ingredients required for testing salt iodine at home?		
**AP23.a**	Salt	Mentioned..................................01<br>Did not mention................................02	☐☐<br>02 ⇨ AP23.c
**AP23.b**	How much Salt	Pinch amount.....................................01<br>Tea spoon full.....................................02<br>Others (please specify)........................77<br>___________________________________<br>Dont know...99<br>Not applicable.....................................88	☐☐
**AP23.c**	Cooked rice	Mentioned..................................01<br>Did not mention.............................02	☐☐<br>02 ⇨ AP23.e
**AP23.d**	How much Cooked rice?	Small amount......................................01<br>5 grains...02<br>Others (please specify).........................77<br>___________________________________<br>Don't know...99<br>Not applicable....................................88	☐☐
**AP23.e**	Lemon juice	Mentioned<br>.....................................01<br>Did not mention.............................02	☐☐<br>02 ⇨ AP24
**AP23.f**	How much Lemon juice?	Few drops...01<br>Others (please specify).........................77<br>___________________________________<br>Don't know...99<br>Not applicable....................................88	☐☐
**AP24**	What colour if salt contains iodine?	Colour does not change<br>...01<br>Blue.. 02<br>Violet...03<br>Others (please<br>specify).............................77	☐☐
**AP25**	What colour if salt does not contain iodine?	Colour does not change<br>...01<br>Blue.. 02	☐☐

		Violet...03<br>Others (please specify)..............................77	
**AP26**	Did you ever see salt being tested by government people in place where you buy your salt from?	Yes……………………………………….........01<br>No……………………………………..........02<br>Don't know...................................99	☐☐

**IS: Use of iodized salt**			
We would like to ask some questions about the salt most commonly used in your household and to take a sample for laboratory testing to see if it is iodized. Please answer all questions below in relation to the salt most commonly used to cook meals in your household.			
	QUESTION	CODING CATEGORIES	CODE
**IS1.**	**Is** the salt currently used to cook meals in your household in the packaging *(such as packets/plastic bags/tins)* you bought it in?	Yes…………………………………..........01<br>No……………………………….............02<br>No salt currently in the household ....03<br>Don't know..99	☐☐
**IS2.**	**Was** the salt currently used to cook meals in your household bought in a sealed package<br><br>*(Check whether the packet is of any brand)*	Yes…………………………………..........01<br>No……………………………….............02<br>Don't know.......................................99	☐☐

**IS3.**	Do you know whether packaged salts have any picture/sign (label) on their packets?	Yes……………………………………………..01<br>No………………………………………………02<br>Don't know......................................99	02⇨IS6<br>99⇨IS6
**IS4.**	If yes, what are the picture/signs that are present on the packet?<br><br>*(Do not prompt)*		
**IS4.a**	BSTI seal	Mentioned...01<br>Did not mention.....................................02	
**IS4.b**	Logo of "iodized salt"	Mentioned...01<br>Did not mention.....................................02	
**IS4.c**	Date of manufacture	Mentioned...01<br>Did not mention.....................................02	
**IS4.d**	Date of Expiry	Mentioned...01<br>Did not mention.....................................02	
**IS4.e**	Weight	Mentioned...01<br>Did not mention.....................................02	
**IS4.f**	Maximum retail price	Mentioned...01<br>Did not mention.....................................02	
**IS4.g**	Name of manufacture	Mentioned...01<br>Did not mention.....................................02	

**IS5.**	*Please look to observe the packet of the salt and record whether the information are present on the packet*	Packet is present......................................01<br>Packet is not present.................................02		02⇨IS6
		BSTI seal	Present=1; Absent=2	
		Logo of "iodized salt"	Present=1; Absent=2	
		Date of manufacture	Present=1; Absent=2	
		Date of Expiry	Present=1; Absent=2	
		Weight	Present=1; Absent=2	
		Maximum retail price	Present=1; Absent=2	
		Name of manufacturer	Present=1; Absent=2	
**IS6**.	Where do you usually buy salts from?	Retailer shops................................. 01<br>Market/haat.............................. 02<br>Hawkers .... 03<br>From ration......................................04<br>Others (please specify........................77		
**IS7**.	When the salt <u>currently used to cook meals</u> in your household was bought, did you look for or ask for iodized salt? (labelled as iodized)	Yes…………………………………….............01<br>No………………………………….….............02<br>Someone else purchased the salt............................03<br>Salt was collected locally at site of production..04		02⇨IS9<br>03⇨IS9<br>04⇨IS9

**IS8**	How did you know it was iodized? (label, packaged)	Labelled as "Iodized".....01<br>Seal of BSTI present.........................02<br>Packaged..................................03<br>The shopkeeper assured.................. 04<br>Can't give a reason........................05	☐☐
**IS9**	Do you use salt for purposes other than cooking or as table salt?<br>*Such as food preservation, feeding livestock or cleaning purpose*	Yes…………………….....01<br>No……………………......02	☐☐<br>02⇨IS11
**IS10**	Please mention the useage of salt in our household other than for cooking, as table salt or for food preservation<br>*Such as feeding livestock or cleaning purpose*		
**IS10.a**	For food preservation	Yes…………………….....01<br>No……………………......02	
**IS10.b**	Feeding livestock/in fish farm	Yes…………………….....01<br>No……………………......02	
**IS10.c**	For cleaning purpose	Yes…………………….....01<br>No……………………......02	
**IS10.d**	Others (Please specify)...........77	Yes…………………….....01<br>No……………………......02	
**IS11**	How much salt do you use for cooking, table salt and preservation in a month?<br>*If the respondent cannot quantify accurately, seek permission to observe the salt container and ask how full it was when salt was purchased and when it was purchased*	.... grams	☐☐☐☐

**IS12.**	On average, what quantity of salt do you buy for consumption (cooking, table salt, preserving foods)?	<100 gms.......................................01<br>100-200 gms.................................02<br>200-400 gms..................................03<br>500 gms..04<br>1 kg...05<br>1-2 kgs......................................06<br>2-4 kgs......................................07<br>>5 kgs.......................................08<br>Don't know...99	☐☐
**IS13.**	Approximately how often do you buy salt for consumption (cooking, table salt, preserving foods)?	More than once a week ....01<br>Every week ....02<br>Two to three times a month......................03<br>Once a month **......................**04<br>Once in 2 to 3 months ....05<br>Once in 4 to 6 months..................06<br>Other ....077<br>Don't Know...99	☐☐
**IS14.**	In the past 7 days, on about how many days did you consume food in the home that was prepared using or seasoned with salt?<br><br>*Such as pickles, fish, others*	No days in this period.............00<br>1 day..01<br>2 days..02<br>3 days ....03<br>4 days..04<br>5 days..05<br>6 days..06<br>7 days..07<br>Don't know..................................99	☐☐<br>00 ⇨PF3<br>99 ⇨PF3
**IS15.**	On days when you use salt, on average how many of your meals in that day are prepared or seasoned with salt?	3 times a day...............................01<br>2 times a day..............................02<br>Once a day..03<br>Don't know..99	☐☐
**IS16.**	How often do you consume food away from home in a week/vendor cooked food at home?	Everyday..01<br>4-6 days.......................................02<br>1-3 days.......................................03<br>No days in this period.......................04<br>Don't know...99	☐☐

**IS17**.	How do you store salt used in your households?<br><br>Seek permission from the respondent to observe the container. Record accordingly. Maximum two codes can be recorded	Plastic container with lid………….01<br>Plastic container without lid ……………...02<br>Clay-made container with lid……………...03<br>Clay-made container without lid………..04<br>Kept in bought packaging..................05<br>Container was not observed................06<br>Record causes..................... 77	☐☐<br><br>☐☐
**IS18.**	Could I please take a sample of your salt for testing of iodine content in a laboratory?	Yes……………………………………………...01<br>No………………………………………………02<br>No salt in household................................03	☐☐<br>02⇨IS23<br>03⇨IS23
**IS19.**	*Collect approximately 50grams of salt from the household into the plastic bag provided and label the sample with the {PSU number} and {household number} on the sticker provided (identical to the number of the front of this questionnaire and in box IS13 below).*<br><br>salt sample was collected...........01<br>salt sample was not collected..........02<br>If 02 go to **FF1**		

**IS20.**	SALT SAMPLE ID<br>1 _ _ _ _ _<br>*Enter the PSU number after 1, followed by the household number*		
**IS21.**	*Conduct a rapid field test from a portion of the salt collected for laboratory test as described in the field manual.*<br>*Enter the number that corresponds to the test outcome.*	Not iodized...........................01<br>Iodized................................02<br>Salt not tested.................03<br>No salt in the home 04	__ __<br>02⇨IS24<br>03⇨IS23
**IS22.**	*The first result was negative. Conduct the retest with recheck solution on a new salt sample, as described in the field manual. Select the code that corresponds to the test outcome.*	Not iodized...........................01<br>Iodized................................02<br>Salt not tested.................03	__ __
**IS23.**	*Please state the reason why the salt was not tested/re-tested*	______________________________<br>______________________________	

**IS24.**	Please provide your valuable feedback to relevant authorities on how to increase public awareness regarding use of iodized salt.		
**IS24.a**	To increase campaign by media	Yes..01<br>No...02	
**IS24.b**	Health workers should be involved more	Yes..01<br>No...02	
**IS24.c**	These topics should be included in school curriculum	Yes..01<br>No...02	
**IS24.d**	Price should be reduced	Yes..01<br>No...02	
**IS24.e**	Salts should be packaged	Yes..01<br>No...02	
**IS24.f**	Packets should be properly labelled	Yes..01<br>No...02	
**IS24.g**	Ensure proper dissemination of information through retailer shops and shopkeepers	Yes..01<br>No...02	
**IS24.h**	Raise public awareness on salt testing for iodine using home ingredients	Yes..01<br>No...02	
**IS24.i**	No advice/could not give any advice	Yes..01<br>No...02	
**IS24.j**	Others (please specify) ______________________________	Yes..01<br>No...02	

**IS25.**	Please provide your valuable feedback on what steps should be taken by relevant authorities to ensure availability of quality iodized salt in market.		
**IS25.a**	Price should be reduced	Yes..01<br>No...02	☐☐
**IS25.b**	Formualte law and ensure proper implementation	Yes..01<br>No...02	☐☐
**IS25.c**	Raise awareness among retailers about salt law	Yes..01<br>No...02	☐☐
**IS25.d**	Formulate law regarding proper packaging and appropriate implementation	Yes..01<br>No...02	☐☐
**IS25.e**	No advice/could not give any advice	Yes..01<br>No...02	☐☐
**IS25.f**	Others (please specify) ___________________________	Yes..01<br>No...02	☐☐

FORTIFIABLE FOODS DATA

Examples of basic contact coverage questions for other fortifiable edible oil
Mark the relevant box with an "√"

FF1: What is the main edible oil consumed by your household
(clarify with: the oil that you use on most days in most meals in the home)?

FF1a:	Soya-bean oil	|__|
FF1b:	Palm oil	|__|
FF1c:	Rice-bran oil	|__| ⇨ **FF4**
FF1d:	Sesame seed	|__| ⇨ **FF4**
FF1e:	Groundnut oil	|__| ⇨ **FF4**
FF1f:	Sunflower oil	|__| ⇨ **FF4**
FF1g:	Mustard seed oil	|__| ⇨ **FF4**
FF1h:	Ghee	|__| ⇨ **FF4**
FF1i:	Butter oil	|__| ⇨ **FF4**
FF1j:	Don't know	|__| ⇨ **FF4**
FF1k:	Other (Please specify)	|__| ⇨ **FF4**

FF2: Can you tell me where you usually get this main edible oil consumed in your household from?
Tick one box only...

FF2a:	Purchased from a store	|__|
FF2b:	Made it at home	|__| ⇨ **FF4**
FF2c:	Food aid	|__| ⇨ **FF4**
FF2d:	Don't know	|__| ⇨ **FF4**
FF2e:	Other (Please specify).............	|__| ⇨ **FF4**

FF3: Can you tell me the brand? *Tick* ***<u>one</u>*** *box only...*

1. (Soybean oil)

FF3a:	Unbranded	|__|
FF3b:	Rupchanda	|__|
FF3c:	Fresh	|__|
FF3d:	Teer	|__|
FF3e:	Pushti	|__|
FF3f:	Sun	|__|
FF3g:	Veola	|__|
FF3h:	Morog	|__|
FF3i:	Mostofa	|__|
FF3j:	Other (Please specify).............	|__|

FF3k:	Could not remember brand name	|__|

2. (Palm oil)

FF3l:	Unbranded	|__|
FF3m:	Meizan	|__|
FF3n:	Lucky	|__|
FF3o:	Fresh	|__|
FF3p:	Family	|__|
FF3q:	Hilsha	|__|
FF3r:	Nurjahan	|__|
FF3s:	Morog	|__|
FF3t:	Mostofa	|__|
FF3u:	Other (Please specify).............	|__|
FF3v:	Could not remember brand name	|__|

FF4: For the most recent major purchase of (main oil type from above)

What was the quantity? |__|__|__|__|ml

Use photos of typical quantities (in plastic bags/bottles) and their grams equivalents to determine the quantity if possible.

FF5: How long does this amount usually last in your household (complete days)?

Number of days: |__|__|__|

Thank the respondent, end the interview and check back that all answers are complete before leaving the household.

Field Notes:

Signature of the interviewer Code | | | |

Signature of the quality controller Code | | | |
