# Supplementary material for: Household coverage of vitamin A fortification of edible oil in Bangladesh
Source: PLoS One. 2019 Apr 3;14(4):e0212257. doi: 10.1371/journal.pone.0212257 (PMC6447147; doi:10.1371/journal.pone.0212257)
Supplement: S2 Table — a—Significant difference in weighted mean oil intake between urban and rural low performing household members (p <0.05). b—Significant difference in weighted mean oil intake between rural other and rural low performing household members (p <0.05). When a superscript is not included in the table means that there was no significant difference between rural low performing and urban or rural others. (DOCX) [file pone.0212257.s002.docx]

**S2 Table: Estimated weighted mean oil (g/d) consumption by strata across age group and gender**

|  |  | **Urban** | | | **Rural Others** | | | **Rural low performing** | | | **Overall** | | |
| --- | --- | --- | --- | --- | --- | --- | --- | --- | --- | --- | --- | --- | --- |
| Age group |  | Weighted N | Mean oil (g/d) | 95% CI | Weighted N | Mean oil (g/d) | 95% CI | Weighted N | Mean oil (g/d) | 95% CI | Weighted N | Mean oil (g/d) | 95% CI |
| 12, 23 months | All | **44** | **9.9^a^** | **8.9, 10.9** | **49** | **7.8** | **6.8, 8.9** | **52** | **7.6** | **6.1, 9.2** | **145** | **8.3** | **7.6, 9.0** |
|  | Male | 17 | 10.1^a^ | 7.8, 12.4 | 28 | 8.7^b^ | 7.3, 10.1 | 22 | 6.8 | 5.6, 8.0 | 67 | 8.6 | 7.6, 9.6 |
|  | Female | 27 | 9.8 | 8.6, 11.1 | 21 | 6.8 | 5.9, 7.7 | 30 | 8.2 | 5.9, 10.4 | 78 | 8.1 | 7.1, 9.0 |
| 24, 59 months | All | **162** | **12^a^** | **11.0, 13.0** | **164** | **10.9^b^** | **9.5, 12.2** | **143** | **7.5** | **5.5, 9.5** | **469** | **10.4** | **9.4, 11.4** |
|  | Male | 83 | 12.3^a^ | 11.0, 13.5 | 83 | 11.6^b^ | 9.8, 13.5 | 63 | 7.6 | 5.2, 10.0 | 229 | 10.8 | 9.4, 12.2 |
|  | Female | 79 | 11.7^a^ | 10.3, 13.2 | 81 | 10.3^b^ | 8.8, 11.7 | 80 | 7.4 | 5.6, 9.2 | 240 | 10.0 | 9.0, 11.0 |
| 5, 14 years | All | **443** | **19.7^a^** | **17.9, 21.5** | **580** | **17.2^b^** | **15.8, 18.7** | **536** | **12.0** | **9.5, 14.5** | **1559** | **16.2** | **14.4, 18.0** |
|  | Male | 228 | 20.3^a^ | 18.3, 22.4 | 293 | 17.6^b^ | 16.2, 19.0 | 288 | 13.3 | 11.0, 15.7 | 809 | 17.0 | 15.5, 18.5 |
|  | Female | 215 | 19^a^ | 17.2, 20.9 | 287 | 16.8^b^ | 15.0, 18.6 | 248 | 10.8 | 8.4, 13.2 | 750 | 15.4 | 13.3, 17.5 |
| 15, 19 years | All | **277** | **28.8^a^** | **26.9, 30.7** | **245** | **26.8^b^** | **23.8, 29.7** | **243** | **18.4** | **16.4, 20.4** | **765** | **25.2** | **22.8, 27.7** |
|  | Male | 126 | 30^a^ | 26.0, 34.0 | 127 | 28.4^b^ | 25.6, 32.2 | 105 | 20.0 | 16.8, 23.2 | 358 | 26.8 | 23.9, 29.8 |
|  | Female | 151 | 27.8^a^ | 23.8, 31.8 | 118 | 25.1^b^ | 21.9, 28.3 | 138 | 17.1 | 15.6, 18.6 | 407 | 23.7 | 21.2, 26.3 |
| 20, 49 years | All | **1065** | **29^a^** | **27.1, 31.0** | **1019** | **26.4^b^** | **24.0, 28.9** | **934** | **20.0** | **17.4, 22.7** | **3018** | **25.6** | **23.8, 27.4** |
|  | Male | 504 | 32.2^a^ | 29.9, 34.4 | 476 | 29.4^b^ | 26.5, 32.4 | 434 | 22.2 | 19.1, 25.3 | 1414 | 28.4 | 26.2, 30.6 |
|  | Female | 561 | 26.3^a^ | 24.6, 28.1 | 543 | 23.9^b^ | 21.8, 26.1 | 500 | 18.0 | 15.6, 20.5 | 1604 | 23.2 | 21.6, 24.8 |
| Over 50 years | All | **312** | **24.8^a^** | **22.8, 26.9** | **357** | **22.8^b^** | **20.7, 24.8** | **381** | **16.9** | **14.5, 19.3** | **1050** | **21.9** | **20.4, 23.4** |
|  | Male | 169 | 26^a^ | 23.6, 28.3 | 186 | 24.1^b^ | 21.6, 26.6 | 209 | 17.3 | 14.5, 20.1 | 564 | 22.9 | 21.1, 24.8 |
|  | Female | 143 | 23.5^a^ | 21.0, 26.0 | 171 | 21.2^b^ | 19.2, 23.1 | 172 | 16.4 | 14.2, 18.6 | 486 | 20.6 | 19.2, 22.0 |

^a^ - Significant difference in weighted mean oil intake between urban and rural low performing household members (p <0.05)

^b^ - Significant difference in weighted mean oil intake between rural other and rural low performing household members (p <0.05)

When a superscript is not included in the table means that there was no significant difference between rural low performing and urban or rural others
