# Supplementary material for: Household coverage of vitamin A fortification of edible oil in Bangladesh
Source: PLoS One. 2019 Apr 3;14(4):e0212257. doi: 10.1371/journal.pone.0212257 (PMC6447147; doi:10.1371/journal.pone.0212257)
Supplement: S3 Table — a—Significant difference in weighted mean oil intake between non-deprived household members with low MPI (MPI <0.33) and deprived household members vulnerable to poverty (MPI ≥ 0.33) (p <0.05). *—Total may not add up because of missing data. When a superscript is not included in the table means that there was no significant difference between non-deprived household members with low MPI and deprived household members with high MPI (MPI ≥ 0.33). (DOCX) [file pone.0212257.s003.docx]

**S3 Table: Estimated weighted mean oil consumption (g/d) by poverty level for different age groups and gender**

|  |  | **Vulnerable to poverty (MPI ≥ 0.33)** | | | **Not vulnerable to poverty (MPI (<0.33)** | | | **Overall** | | |
| --- | --- | --- | --- | --- | --- | --- | --- | --- | --- | --- |
| Age group | Gender | Weighted N* | Mean oil (g/d) | 95% CI | Weighted N* | Mean oil (g/d) | 95% CI | Weighted N* | Mean oil (g/d) | 95% CI |
| 12 - 23 months | All | **55** | **6.7^a^** | **5.7, 7.7** | **90** | **9.3** | **8.2, 10.3** | **145** | **8.3** | **7.6, 9.0** |
|  | Male | 22 | 7.2^a^ | 5.5, 9.0 | 45 | 9.2 | 8.2, 10.2 | 67 | 8.6 | 7.6, 9.6 |
|  | Female | 33 | 6.4 | 5.4, 7.4 | 45 | 9.3 | 7.5, 11.1 | 78 | 8.1 | 7.1, 9.0 |
| 24 - 59 months | All | **177** | **8.7^a^** | **7.0, 10.4** | **283** | **11.6** | **10.6, 12.5** | **469** | **10.4** | **9.4, 11.4** |
|  | Male | 94 | 9.3 | 7.2, 11.5 | 130 | 12.1 | 10.6, 13.7 | 229 | 10.8 | 9.4, 12.2 |
|  | Female | 83 | 8.0^a^ | 6.3, 9.7 | 153 | 11.1 | 10.1, 12.2 | 240 | 10.0 | 9.0, 11.0 |
| 5 - 14 years | All | **855** | **14.3^a^** | **12.1, 16.6** | **678** | **19.1** | **17.9, 20.2** | **1559** | **16.2** | **14.4, 18.0** |
|  | Male | 454 | 15.1^a^ | 13.1, 17.1 | 342 | 19.8 | 18.3, 21.2 | 809 | 17.0 | 15.5, 18.5 |
|  | Female | 401 | 13.5^a^ | 10.8, 16.2 | 336 | 18.3 | 16.9, 19.8 | 750 | 15.4 | 13.3, 17.5 |
| 15 - 19 years | All | **236** | **21.5^a^** | **18.6, 24.4** | **516** | **27.5** | **25.3, 29.7** | **765** | **25.2** | **22.8, 27.7** |
|  | Male | 116 | 24.0^a^ | 19.4, 28.6 | 239 | 28.7 | 26.0, 31.4 | 358 | 26.8 | 23.9, 29.8 |
|  | Female | 120 | 18.5^a^ | 16.4, 20.7 | 277 | 26.4 | 23.3, 29.5 | 407 | 23.7 | 21.2, 26.3 |
| 20 - 49 years | All | **1065** | **21.9^a^** | **19.8, 24.0** | **1881** | **28.0** | **26.3, 29.7** | **3018** | **25.6** | **23.8, 27.4** |
|  | Male | 484 | 24.3^a^ | 21.8, 26.7 | 891 | 30.9 | 28.7, 33.1 | 1414 | 28.4 | 26.2, 30.6 |
|  | Female | 581 | 19.9^a^ | 17.8, 21.9 | 990 | 25.4 | 23.9, 26.9 | 1604 | 23.2 | 21.6, 24.8 |
| Over 50 years | All | **315** | **19.4^a^** | **16.8, 21.9** | **679** | **23.4** | **21.7, 25.0** | **1050** | **21.9** | **20.4, 23.4** |
|  | Male | 169 | 20.6^a^ | 17.4, 23.8 | 370 | 24.2 | 22.3, 26.2 | 564 | 22.9 | 21.1, 24.8 |
|  | Female | 146 | 18.0^a^ | 15.8, 20.2 | 309 | 22.3 | 20.5, 24.0 | 486 | 20.6 | 19.2, 22.0 |

^a^ - Significant difference in weighted mean oil intake between household members not vulnerable to poverty (MPI <0.33) and household members vulnerable to poverty (MPI ≥ 0.33) (p <0.05)

* - Total may not add up because of missing data

When a superscript is not included in the table means that there was no significant difference between household members not vulnerable to poverty (MPI <0.33) and household members vulnerable to poverty (MPI ≥ 0.33)
