# Supplementary material for: Household coverage of vitamin A fortification of edible oil in Bangladesh
Source: PLoS One. 2019 Apr 3;14(4):e0212257. doi: 10.1371/journal.pone.0212257 (PMC6447147; doi:10.1371/journal.pone.0212257)
Supplement: S4 Table — M–Male; F–Female. a—Significant difference in percentage vitamin A EAR (weighted) met by consuming vitamin A fortified edible oil when comparing urban and rural low performing household members (p <0.05). b—Significant difference in percentage vitamin A EAR (weighted) met by consuming vitamin A fortified edible oil when comparing rural other and rural low performing household members (p <0.05). c–Weighted mean. When a superscript is not included in the table, it means that there is no significant difference between rural low performing and urban or rural others (DOCX) [file pone.0212257.s004.docx]

**S4 Table: Daily vitamin A EAR (%) expected to be met by consuming edible oil fortified at different levels of vitamin A (15 μg/g RE, 20 μg/g RE, 30 μg/g RE), stratified by strata, age group and gender**

|  |  | **Urban** | | | | **Rural low performing** | | | | **Rural Others** | | | |
| --- | --- | --- | --- | --- | --- | --- | --- | --- | --- | --- | --- | --- | --- |
| Age range |  | **N** | **15 μg/g RE^c^** | **20 μg/g RE^c^** | **30 μg/g RE^c^** | **N** | **15 μg/g RE^c^** | **20 μg/g RE^c^** | **30 μg/g RE^c^** | **N** | **15 μg/g RE^c^** | **20 μg/g RE^c^** | **30 μg/g RE^c^** |
| 12 - 23 mo (%) | All | **44** | **33.9^a^ (30.5, 37.3)** | **45.2^a^ (40.6, 49.7)** | **67.8^a^ (61.0, 74.6)** | **52** | **26.0 (20.6, 31.4)** | **34.7 (27.5, 41.9)** | **52.1 (41.3, 62.9)** | **49** | **26.7 (23.1, 30.4)** | **35.6 (30.7, 40.5)** | **53.5 (46.1, 60.8)** |
|  | M | 17 | 34.5^a^ (26.7, 42.3) | 46.1^a^ (35.7, 46.5) | 69.1^a^ (53.5, 84.7) | 22 | 23.2 (19.1, 27.3) | 30.9 (25.5, 36.4) | 46.4 (38.2, 54.5) | 28 | 29.7^b^ (25.0, 34.4) | 39.6^b^ (33.3, 45.9) | 59.4^b^ (49.9, 68.8) |
|  | F | 27 | 33.6 (29.4, 37.7) | 44.8 (39.2, 50.3) | 67.1 (58.9, 75.4) | 30 | 27.8 (20.1, 35.6) | 37.1 (26.8, 47.4) | 55.7 (40.2, 71.1) | 21 | 23.3 (20.1, 26.4) | 31.0 (26.8, 35.2) | 46.5 (40.2, 52.8) |
| 24 - 59 mo (%) | All | **162** | **38.5^a^ (35.2, 41.8)** | **51.3^a^ (47.0, 55.5)** | **77.0^a^ (70.4, 83.5)** | **143** | **24.0 (17.5, 30.4)** | **32.0 (23.4, 40.6)** | **47.9 (35.0, 60.8)** | **164** | **34.9^b^ (30.6, 39.2)** | **46.5^b^ (40.8, 52.3)** | **69.8^b^ (61.1, 78.4)** |
|  | M | 83 | 39.3^a^ (35.4, 43.2) | 52.4^a^ (47.2, 57.6) | 78.6^a^ (70.8, 86.5) | 63 | 24.3 (16.5, 32.1) | 32.4 (22.0, 42.8) | 48.6 (33.1, 64.2) | 83 | 37.3^b^ (31.5, 43.2) | 49.8^b^ (41.9, 57.6) | 74.7^b^ (62.9, 86.5) |
|  | F | 79 | 37.7^a^ (33.1, 42.2) | 50.2^a^ (44.2, 56.3) | 75.4^a^ (66.3, 84.4) | 80 | 23.6 (17.8, 29.4) | 31.5 (23.8, 39.2) | 47.3 (35.7, 58.9) | 81 | 32.9^b^ (28.3, 37.6) | 43.9^b^ (37.7, 50.1) | 65.9^b^ (56.6, 75.2) |
| 5 - 14 y (%) | All | **443** | **52.1^a^ (47.4, 56.8)** | **69.5^a^ (63.63, 75.4)** | **104.2^a^ (94.8, 113.6)** | **536** | **31.8 (25.2, 38.5)** | **42.4 (33.6, 51.3)** | **63.7 (50.4, 76.9)** | **580** | **45.5^b^ (41.7, 49.3)** | **60.6^b^ (55.5, 65.7)** | **91.0^b^ (83.3, 98.6)** |
|  | M | 228 | 53.8^a^ (48.4, 59.1) | 71.7^a^ (64.6, 78.8) | 107.5^a^ (96.9, 118.2) | 288 | 35.3 (29.1, 41.4) | 47.0 (38.8, 55.3) | 70.5 (58.2, 82.9) | 293 | 46.6^b^ (42.9, 50.2) | 62.1^b^ (57.2, 66.9) | 93.1^b^ (85.9, 100.4) |
|  | F | 215 | 50.3^a^ (45.4, 55.2) | 67.1^a^ (60.6, 73.6) | 100.6^a^ (90.8, 110.4) | 248 | 28.5 (22.1, 34.9) | 38.0 (29.4, 46.5) | 57.0 (44.1, 69.8) | 287 | 44.3^b^ (39.6, 49.0) | 59.1^b^ (52.8, 65.4) | 88.6^b^ (79.2, 98.0) |
| 15 - 19 y (%) | All | **277** | **65.5^a^ (61.1, 69.9)** | **87.3^a^ (81.5, 93.2)** | **131.0^a^ (122.3, 139.8)** | **243** | **41.8 (37.3, 46.4)** | **55.8 (49.7, 61.9)** | **83.7 (74.5, 92.9)** | **245** | **60.8^b^ (54.1, 67.5)** | **81.1^b^ (72.1, 90.0)** | **121.6^b^ (108.2, 135.1)** |
|  | M | 126 | 68.2^a^ (59.2, 77.2) | 90.9^a^ (78.9, 102.9) | 136.4^a^ (118.3, 154.4) | 105 | 45.5 (38.2, 52.7) | 60.6 (51.0, 70.3) | 90.9 (76.4, 105.4) | 127 | 64.5^b^ (55.8, 73.1) | 86.0^b^ (74.4, 97.5) | 129.0^b^ (111.6, 146.3) |
|  | F | 151 | 63.2^a^ (54.2, 72.3) | 84.3^a^ (72.2, 96.3) | 126.4^a^ (108.3, 144.5) | 138 | 38.9 (35.5, 42.4) | 51.9 (47.3, 56.5) | 77.8 (70.9, 84.8) | 118 | 57.0^b^ (49.7, 64.3) | 76.0^b^ (66.3, 85.7) | 114.0^b^ (99.4, 128.6) |
| 20 - 49 y (%) | All | **1065** | **72.4^a^ (67.7, 77.1)** | **96.6^a^ (90.3, 102.9)** | **144.9^a^ (135.5, 154.3)** | **934** | **49.8 (43.1, 56.6)** | **66.5 (57.5, 75.4)** | **99.7 (86.2, 113.2)** | **1019** | **66.0^b^ (59.9, 72.2)** | **88.1^b^ (79.9, 96.2)** | **132.1^b^ (119.9, 144.3)** |
|  | M | 504 | 73.1^a^ (68.0, 78.1) | 97.5^a^ (90.7, 104.2) | 146.2^a^ (136.1, 156.3) | 434 | 50.5 (43.4, 57.5) | 67.3 (57.9, 76.7) | 101.0 (86.9, 115.0) | 476 | 66.9^b^ (60.2, 73.6) | 89.2^b^ (80.3, 98.1) | 133.8^b^ (120.5, 147.1) |
|  | F | 561 | 71.9^a^ (67.1, 76.6) | 95.8^a^ (89.5, 102.1) | 143.7^a^ (134.2, 153.3) | 500 | 49.3 (42.6, 55.9) | 65.7 (56.9, 74.5) | 98.5 (85.3, 111.8) | 543 | 65.3^b^ (59.4, 71.3) | 87.1^b^ (79.2, 95.0) | 130.7^b^ (118.8, 142.5) |
| Over 50 y (%) | All | **312** | **56.4^a^ (51.8, 61.1)** | **75.3^a^ (69.1, 81.4)** | **112.9^a^ (103.6, 122.2)** | **381** | **38.4 (33.0, 43.8)** | **51.2 (44.0, 58.4)** | **76.8 (66.0, 87.5)** | **357** | **51.7^b^ (47.1, 56.3)** | **69.0^b^ (62.8, 75.1)** | **103.4^b^ (94.2, 112.7)** |
|  | M | 169 | 59.0^a^ (53.6, 64.4) | 78.7^a^ (71.5, 85.9) | 118.0^a^ (107.3, 128.8) | 209 | 39.3 (32.9, 45.6) | 52.3 (43.9, 60.8) | 78.5 (65.9, 91.2) | 186 | 54.8^b^ (49.9, 61.9) | 73.1^b^ (65.6, 80.7) | 109.7^b^ (98.4, 121.0) |
|  | F | 143 | 53.3^a^ (47.6, 59.0) | 71.1^a^ (63.5, 78.7) | 106.6^a^ (95.2, 118.0) | 172 | 37.3 (32.3, 42.3) | 49.7 (43.0, 56.5) | 74.6 (64.5, 84.7) | 171 | 48.1^b^ (43.7, 52.6) | 64.2^b^ (58.3, 70.1) | 96.3^b^ (87.4, 105.2) |

M – Male; F - Female

^a^ - Significant difference in percentage vitamin A EAR (weighted) met by consuming vitamin A fortified edible oil when comparing urban and rural low performing household members (p <0.05)

^b^ - Significant difference in percentage vitamin A EAR (weighted) met by consuming vitamin A fortified edible oil when comparing rural other and rural low performing household members (p <0.05)

^c^ – Weighted mean

When a superscript is not included in the table, it means that there is no significant difference between rural low performing and urban or rural others
