# Supplementary material for: Household coverage of vitamin A fortification of edible oil in Bangladesh
Source: PLoS One. 2019 Apr 3;14(4):e0212257. doi: 10.1371/journal.pone.0212257 (PMC6447147; doi:10.1371/journal.pone.0212257)
Supplement: S5 Table — a—Significant difference in percentage vitamin EAR (weighted) met by consuming vitamin A fortified edible oil between those household members, categorized by poverty status, based on households with high MPI (in poverty) and low MPI (not in poverty) (MPI <0.33) (p <0.05). b–Weighted mean. No superscript indicates that that there is no significant difference between household members between low MPI (not in poverty) and high MPI (in poverty). (DOCX) [file pone.0212257.s005.docx]

**S5 Table: Daily vitamin A EAR (%) expected to be met by consuming edible oil fortified at different levels of vitamin A (15 μg/g RE, 20 μg/g RE, 30 μg/g RE), stratified by age group, gender and poverty level**

|  |  | **Poor (MPI ≥ 0.33) ^b^** | | | | **Not poor (MPI (<0.33) ^b^** | | | | Overall | | | |
| --- | --- | --- | --- | --- | --- | --- | --- | --- | --- | --- | --- | --- | --- |
| Age range |  | N | 15 μg/g RE | 20 μg/g RE | 30 μg/g RE | N | 15 μg/g RE | 20 μg/g RE | 30 μg/g RE | N | 15 μg/g RE | 20 μg/g RE | 30 μg/g RE |
| 12 - 23 mo | All | 55 | 22.9 (19.5, 26.3) | 30.6 (26.0, 35.1) | 45.8 (39.0, 52.6) | 90 | 31.5^a^ (27.4, 34.7) | 42.1^a^ (36.5, 46.2) | 63.1^a^ (56.1, 70.1) | 145 | 28.3 (25.9, 30.7) | 38.0 (34.5, 41.0) | 56.6 (51.8, 61.5) |
|  | Male | 22 | 24.6 (18.6, 30.7) | 32.9 (24.9, 40.9) | 49.3 (37.3, 61.3) | 45 | 31.3 (27.9, 34.8) | 41.8 (37.2, 46.4) | 62.7 (55.8, 69.6) | 67 | 29.3  (25.8, 32.7) | 39.0 (34.4, 43.7) | 58.5 (51.6, 65.5) |
|  | Female | 33 | 21.9 (18.4, 25.3) | 29.2 (24.6, 33.8) | 43.7 (36.8, 50.6) | 45 | 31.7^a^ (25.5, 38.0) | 42.3^a^ (34.1, 50.6) | 63.5^a^ (51.1, 75.9) | 78 | 27.5 (24.3, 30.8) | 36.7 (32.3, 41.0) | 55.1 (48.6, 61.5) |
| 24 - 59 mo | All | 177 | 27.7 (22.7, 32.8) | 37.0 (30.2, 43.7) | 55.5 (45.3, 65.6) | 283 | 36.7^a^ (33.8, 40.1) | 48.9^a^ (45.1, 53.5) | 73.4^a^ (67.2, 79.6) | 469 | 33.3 (30.1, 36.6) | 44.4 (40.1, 48.8) | 66.7 (60.2, 73.1) |
|  | Male | 94 | 29.5 (23.1, 35.9) | 39.3 (30.7, 47.9) | 59.0 (46.1, 71.9) | 130 | 38.1^a^ (33.2, 43.1) | 50.9^a^ (44.2, 57.5) | 76.3^a^ (66.4, 86.2) | 229 | 34.8 (30.3, 39.3) | 46.4 (40.4, 52.3) | 69.5 (60.5, 78.5) |
|  | Female | 83 | 25.9 (20.7, 31.2) | 34.5 (27.6, 41.5) | 51.8 (41.3, 62.3) | 153 | 35.5^a^ (32.3, 38.8) | 47.4^a^ (43.0, 51.8) | 71.1^a^ (64.5, 77.7) | 240 | 32.1 (28.9, 35.2) | 42.8 (38.6, 46.9) | 64.1 (57.9, 70.4) |
| 5 - 14 y | All | 855 | 37.9 (32.0, 43.7) | 50.5 (42.7, 58.3) | 75.7 (65.1, 87.4) | 678 | 49.9^a^ (47.1, 53.1) | 66.5^a^ (62.8, 70.8) | 99.8^a^ (93.5, 106.2) | 1559 | 42.8 (38.1, 47.5) | 57.0 (50.8, 63.3) | 85.6 (76.1, 95.0) |
|  | Male | 454 | 40.0 (34.8, 45.1) | 53.3 (46.4, 60.2) | 79.9 (69.7, 90.2) | 342 | 51.9^a^ (48.1, 55.7) | 69.2^a^ (64.1, 74.2) | 103.8^a^ (96.2, 111.4) | 809 | 44.8 (40.8, 48.8) | 59.7 (54.4, 65.0) | 89.6 (81.7, 97.5) |
|  | Female | 401 | 35.7 (28.8, 42.5) | 47.5 (38.4, 56.7) | 71.3 (57.6, 85.1) | 336 | 47.8^a^ (43.9, 51.7) | 63.8^a^ (58.6, 68.9) | 95.6^a^ (87.9, 103.4) | 750 | 40.6 (35.0, 46.2) | 54.2 (46.7, 61.7) | 81.3 (70.1, 92.5) |
| 15 - 19 y | All | 236 | 48.8 (42.5, 55.1) | 65.5 (56.6, 73.5) | 97.6 (84.9, 110.2) | 516 | 62.1^a^ (57.2, 67.1) | 82.9^a^ (76.5, 90.5) | 124.3^a^ (114.5, 134.1) | 765 | 57.3 (51.7, 62.9) | 76.4 (69.0, 83.8) | 114.6 (103.5, 125.7) |
|  | Male | 116 | 54.7 (44.4, 65.1) | 73.0 (59.2, 86.8) | 109.5 (88.8, 130.2) | 239 | 65.3 (59.2, 71.4) | 87.1 (79.0, 95.2) | 130.6 (118.5, 142.8) | 358 | 61.0 (54.3, 67.7) | 81.3 (72.4, 90.2) | 122.0 (108.6, 135.4) |
|  | Female | 120 | 42.2 (37.5, 47.0) | 56.3 (50.0, 62.6) | 84.4 (75.0, 93.9) | 277 | 59.5^a^ (52.5, 66.6) | 79.4^a^ (70.0, 88.8) | 119.1^a^ (105.0, 133.2) | 407 | 53.9 (48.1, 59.7) | 71.8 (64.1, 79.6) | 107.7 (96.2, 119.3) |
| 20- 49 y | All | 1065 | 55.3 (50.0, 60.5) | 73.7 (66.7, 80.7) | 110.5 (100.1, 121.0) | 1881 | 69.6^a^ (65.3, 73.9) | 92.8^a^ (87.3, 99.1) | 139.2^a^ (130.7, 147.7) | 3018 | 63.9 (59.3, 68.5) | 85.2 (79.1, 91.4) | 127.8 (118.6, 137.1) |
|  | Male | 484 | 56.0 (50.7, 61.3) | 74.6 (67.6, 81.7) | 111.9 (101.3, 122.5) | 891 | 70.1^a^ (65.1, 75.0) | 93.4^a^ (86.8, 100.0) | 140.2^a^ (130.3, 150.1) | 1414 | 64.6 (59.6, 69.6) | 86.1 (79.5, 92.7) | 129.1 (119.2, 139.1) |
|  | Female | 581 | 54.7 (49.3, 60.0) | 72.9 (65.8, 80.0) | 109.4 (98.7, 120.1) | 990 | 69.2^a^ (65.1, 73.2) | 92.2^a^ (86.8, 97.6) | 138.3^a^ (130.3, 146.4) | 1604 | 63.4 (58.9, 67.8) | 84.5 (78.5, 90.4) | 126.7 (117.8, 135.6) |
| Over 50 y | All | 315 | 44.2 (39.0, 49.4) | 58.9 (52.0, 65.8) | 88.4 (78.0, 98.8) | 679 | 52.5^a^ (48.9, 56.1) | 70.0^a^ (66.2, 75.7) | 105.1^a^ (97.9, 112.2) | 1050 | 49.7 (46.3, 53.1) | 66.3 (61.8, 70.8) | 99.4 (92.6, 106.1) |
|  | Male | 169 | 46.8 (40.2, 53.3) | 62.3 (53.6 , 71.0) | 93.5 (80.4, 106.6) | 370 | 54.6^a^ (50.2, 58.9) | 72.7^a^ (66.9, 78.5) | 109.1^a^ (100.4, 117.8) | 564 | 52.1 (47.9, 56.3) | 69.4 (63.9, 75.0) | 104.2 (95.8, 112.5) |
|  | Female | 146 | 41.5 (37.0, 46.0) | 55.4 (49.4, 61.4) | 83.1 (74.1, 92.1) | 309 | 50.0^a^ (46.3, 53.7) | 66.7^a^ (61.8, 71.6) | 100.1^a^ (92.7, 107.5) | 486 | 46.9 (43.7, 50.0) | 62.5 (58.3, 66.7) | 93.7 (87.4, 100.1) |

^a^ - Significant difference in percentage vitamin EAR (weighted) met by consuming vitamin A fortified edible oil between those household members, categorized by poverty status, based on households with high MPI (deprived) and low MPI (non-deprived) (MPI <0.33) (p <0.05)

^b^ – Weighted mean

No superscript indicates that that there is no significant difference between household members between low MPI (deprived) and high MPI (non-deprived) households
